# Lipid Class Prediction from MS1 Data using Gaussian Graphical Models

**DOI:** 10.1021/acs.analchem.5c08067

**Published:** 2026-05-20

**Authors:** Thomas Rix, Caroline Jane Sands, Alma Villaseñor, Ana Gradillas, Coral Barbas, Elena Chekmeneva, Elizabeth J Want, Timothy M D Ebbels

**Affiliations:** † Section of Bioinformatics, Division of Systems Medicine, Department of Metabolism, Digestion and Reproduction, Faculty of Medicine, 4615 Imperial College London, London W12 0NN, U.K.; ‡ National Phenome Centre, Department of Metabolism, Digestion and Reproduction, Imperial College London, London W12 0NN, U.K.; ¶ Centro de Metabolómica y Bioanálisis (CEMBIO), Facultad de Farmacia, Universidad San Pablo-CEU, 16345 CEU Universities, Urbanización Monteprıncipe, Boadilla del Monte 28660, Spain; § Departamento de Ciencias Médicas Básicas, Instituto de Medicina Molecular Aplicada (IMMA) Nemesio Dıez, Facultad de Medicina, 202083 Universidad San Pablo-CEU, CEU Universities, Boadilla del Monte 28668, Spain; ∥ Section of Bioanalytical Chemistry, Division of Systems Medicine, Department of Metabolism, Digestion and Reproduction, Faculty of Medicine, Imperial College London, London W12 0NN, U.K.

## Abstract

Liquid chromatography–mass spectrometry (LC-MS) untargeted analysis enables comprehensive lipid profiling of biological samples. However, system-level interpretation is often limited by the large number of unannotated features. Assigning features to lipid classes provides a higher-level, yet informative, overview that complements detailed structural analysis and supports biological interpretation at the class level. Recent advances in the systematic prediction of chemical class using tandem mass spectrometry (MS2) help address this; however, a substantial proportion of features in untargeted LC-MS data sets are typically characterized only at the MS1 level. Here, we present a workflow to systematically predict the lipid class from MS1-only data in untargeted LC-MS, without requiring prior annotations or MS2. Motivated by previous research showing that Gaussian graphical models (GGMs) estimated from feature intensities can encode the lipid class structure, our method, GgmLipidClassifier (GLC), combines conventional accurate-mass database searching with a GGM-derived network structure in a unified scoring framework to predict lipid class according to the LIPID MAPS Structure Database (LMSD) ontology. Across three human serum and plasma data sets, GLC achieved overall accuracies of 82–90% at the LMSD main class-level and 72–86% at the lipid subclass level, with improved accuracy and reduced uncertainty compared to closest-*m*/*z* matching. GLC provides class predictions for most detected features and also generates prediction quality scores to support downstream interpretation. Applied to serum samples from an Alzheimer’s disease study, lipid class enrichment based on GLC predictions was highly consistent with class enrichment derived from ground-truth lipid annotations. Importantly, GLC extended coverage to classes missing from the annotation set, revealing biologically plausible associations with Alzheimer’s disease, including cholesterol and derivatives, vitamin D_3_ and derivatives, and plasmalogen glycerophosphoethanolamines. Overall, GLC provides robust lipid class predictions from MS1-only data, generating lipid class assignments for most detected features and complementing conventional analysis to support broader system-level interpretation.

## Graphical abstract

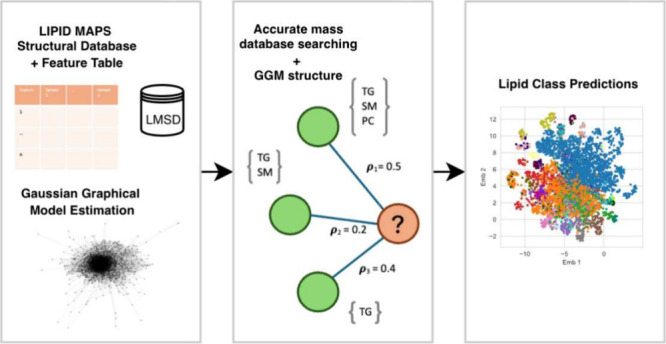

## Introduction

The comprehensive characterization of the lipidome, the full set of lipids and their abundance profiles within a biological system, has become increasingly important for understanding biochemical processes associated with disease.^1,2^ Liquid chromatography coupled with mass spectrometry (LC-MS) is widely considered the technique of choice, primarily due to its high sensitivity.^3^ However, the systems-level interpretation of the lipidome is often limited by the sparsity of annotated lipid species in untargeted LC-MS lipidomics workflows.^4^ The difficulties of lipid annotation are manifold, yet it is an essential step in drawing biological insight from the data. Features are characterized by a mass-to-charge ratio (*m*/*z*) and retention time (rt), and while hundreds to thousands of features are often detected, multiple potential database matches frequently occur due to the formation of different ion types and the high isomeric and isobaric complexity of the lipidome.^5,6^ Fragmentation spectra from tandem mass spectrometry (MS/MS or MS2) can be matched to reference MS2 spectral libraries to facilitate annotation, but most spectra cannot be confidently assigned to specific structures.^7^ Furthermore, MS2 coverage is often low and corresponds to a small fraction of detected MS1 features in a sample set.^8,9^ Ultimately, lipid annotation is still a time-consuming and labor-intensive process, which limits the biological insights that can be drawn from the data.

Recent advancements in the systematic prediction of lipid class from MS2 data provide a higher level yet informative descriptor of lipid structure. These approaches are characterized by their ability to assign chemical class predictions systematically to detected spectra, including those not represented in databases. Methods such as CANOPUS^10^ for general compound class classification and MS2Lipid^11^ specifically designed for the LIPID MAPS Structural Database (LMSD) class prediction are highly accurate, with MS2Lipid achieving over 89.2% accuracy on external validation data sets for LMSD subclass. By predicting classes for most detected spectra, these methods surpass the typical coverage available from more structurally specific annotations and enable system-level interpretation at the class level. However, data-dependent acquisition (DDA) enables fragmentation of only the most abundant ions, and while estimates vary and are dependent on sample, platform, and protocol, MS2 data are not acquired for most features.^8,9^ Therefore, it is necessary to predict the lipid class for features detected only at the MS1 level to uncover greater system-level insights and facilitate structural annotation efforts for low-abundance features. This challenge has been partially addressed by propagating structural information from ion forms with MS2 data to those without, as demonstrated by approaches such as ion identity molecular networking (IIMN)^12^ and consensus classifications of *in silico* elucidations (ConCISE).^13^ In addition, tentative propagation to MS1 features based on mass differences corresponding to metabolic reactions was explored in a case study investigating the herbivore-induced metabolic response to corn silk.^14^ Despite these advances, a substantial number of features remain inaccessible due to their dependence on the availability of MS2 data.

To the best of our knowledge, no existing approach directly predicts lipid class from MS1 data alone. Although there are numerous systematic *in silico* tools for metabolite annotation, they are typically designed to predict more detailed molecular structures. However, class-level analysis is possible in principle by mapping predicted structures to class according to a chemical class ontology. These include approaches that consider different network layers of metabolite information, starting from ‘known’ seed annotations and propagating to ‘unknown’ features.^15–17^ However, for the comprehensive characterization of MS1 features, the effectiveness of these approaches is heavily dependent on the coverage and diversity of seed metabolites, which are often limited in both number and chemical space. An alternative tentative annotation strategy involves modeling both the multiple feature-metabolite matches and network of metabolite relations, typically a reaction network, under the assumption that features associated with a biological outcome will colocalize.^18–20^ However, this approach excludes structures that are not curated. Additionally, these methods are designed for annotating perturbed features and may underperform for low-effect-size lipids. In summary, existing approaches for metabolite annotation are not intended for systematic lipidome-wide coverage from MS1 data, and neither are they explicitly designed for class analysis.

Gaussian graphical models (GGMs) have a long-standing role in the interpretation of metabolomic data sets,^21–23^ where they model conditional dependencies between metabolite intensities using partial correlations. By filtering out indirect associations, GGMs reduce spurious correlations and produce sparser, more interpretable networks than Pearson correlation approaches. In a targeted lipidomics study, Krumsiek et al.^21^ showed that GGM modularity reflects lipid class organization, while Dakic et al.^24^ extended these findings to several hundred annotations in untargeted lipid data sets, demonstrating that lipid classes tend to cluster within GGM community structures across hundreds of annotations. Collectively, these studies suggest that GGM structure provides useful information to facilitate lipid class prediction.

On the basis of these observations, we developed GgmLipidClassifier (GLC), a workflow that integrates conventional accurate-mass database searching with GGM structure to predict lipid classes directly from MS1 data. By leveraging the conditional dependencies encoded in GGM, GLC captures the tendency of lipids to cluster by class, enabling systematic class-level prediction without requiring MS2 or seed annotations. This approach provides robust class predictions validated against ground-truth annotations across three human serum and plasma data sets, offers quality-scored predictions for most detected features, and expands class-level coverage beyond the scope of existing annotations to enable system-wide interpretation of the lipidome.

## Methods

### Data Sets

Three publicly available reversed-phase (RP) LC-MS assays were used. Each assay was acquired and processed following the established and validated workflows of the originating institutions. Lipid class predictions were generated using the feature table as input, which included *m*/*z*, rt, and sample intensities for each detected feature. These predictions were made using either the ungrouped feature table (each feature is an ion) or the grouped feature table containing deconvoluted features. For example, an analyte with ion forms [M + H]^+^ and [M + Na]^+^ would appear as two separate features in the ungrouped table but would be deconvoluted into a single representative feature in the grouped table. Ground truth used in model evaluation was provided as annotations from targeted feature extraction using the institution’s in-house database. Briefly, the data sets are summarized below.

As part of the AddNeuroMed biomarker discovery study,^25^ 613 serum samples were profiled at the UK National Phenome Centre using untargeted LC-MS with a lipidomic RP column in ESI(±) mode on a Waters Xevo G2-S Quadrupole Time-of-Flight (QTOF) mass spectrometer, following Lewis et al.^26^ The two ionization modes were processed and analyzed separately and are referred to as the AddNeuroMed-LPOS and AddNeuroMed-LNEG data sets. The cohort included 173 Alzheimer’s disease (AD) cases, 270 mild cognitive impairment (MCI) participants, and 170 controls; sex and age were recorded as covariates (see Table S1). All samples were analyzed in ESI­(+), and 479 samples were analyzed in ESI(−). Raw files were converted to mzML using ProteoWizard^27^ and preprocessed with XCMS v4.3.0 ^28,29^ and nPYc-toolbox v1.2.7,^30^ yielding 4,886 LPOS and 2,091 LNEG features. Targeted feature extraction (outlined in section S.1.1) using PeakPantheR^31^ provided ground-truth annotations for 183 positive-mode and 56 negative-mode LMSD subclass-specific annotations that could be mapped to the feature table (section S.1.2).

The metformin high-intensity interval exercise (metformin-HIIE) assay consisted of nine healthy male participants. Full inclusion criteria, study design, lipidomic profiling, and preprocessing are described in previous publications^32,33^ and outlined in section S.1.3. Briefly, venous blood was collected at 6 predefined time points during the resting session (metformin only) and 14 predefined time points during the exercise session (high-intensity interval exercise (HIIE) following metformin intake), resulting in 20 samples per participant and 180 plasma samples in total. Samples were analyzed using an Agilent 1290 Infinity II UHPLC system coupled to an Agilent 6545 QTOF Mass Spectrometer (Agilent Technologies, Santa Clara, USA) equipped with a Dual Agilent Jet Stream Electrospray source (Dual AJS ESI). For the present analysis, only the ESI­(+) assay was used. Untargeted MS1 raw Agilent files were preprocessed using the MassHunter Profinder software (version B.10.0.2, Agilent Technologies, Santa Clara, CA, USA), which included a feature deconvolution step yielding a feature table of 1,171 entries (see section S.1.3 for further details). Additionally, high confidence lipid annotations were obtained using a Human Plasma NIST SRM 1950 lipodomic database as a template to perform a protocol described by Martınez et al.,^34^ resulting in 131 features mapping to the feature table with lipid subclass specificity (section S.1.2).

### GGM Estimation

From an assay’s feature table, intensity values for features across samples were used to estimate a GGM using the GeneNet package.^35,36^ In the resulting network, each node represents a feature, and edges correspond to partial correlations between features that are significant after GeneNet’s multiple hypothesis testing procedure (α = 0.05). Only features within the main-subgraph of the GGM were used in downstream analysis, which covered 99.81% of features in AddNeuroMed-LPOS, 99.62% in AddNeuroMed-LNEG, and 93.08% in metformin-HIIE. Prior to network inference, features with more than ten percent missing values were excluded. Intensity values were log-transformed, and remaining missing values were imputed using scikit-learn’s^37^ KNNImputer with default parameters.

### Tentative Mapping of Features to Structures

Prior to lipid class prediction, features were tentatively mapped to structures in the LIPID MAPS Structural Database (LMSD) (downloaded on the 14th of March 2024). For the metformin-HIIE assay, this was performed by matching the neutral mass of each feature to the monoisotopic mass of LMSD entries within a ±5 ppm tolerance, as the preprocessing workflow includes feature grouping. For the AddNeuroMed assays, where features were not grouped, ion forms were explicitly considered during matching. To minimize false positives, each feature was searched as [M + H]^+^ mode under ESI­(+) or [M – H]^−^ under ESI(−). In addition, plausible ion types were inferred using a kernel density estimation approach based on the *m*/*z* differences of features within a one-second rt window, similar to the method described in^38,39^ and described in S.1.4. Furthermore, to exclude inconsistent matches, we applied a simple rt–logP monotonic regression filter, described in section S.1.5.

### Class Scoring

GLC predicts the lipid subclass of a feature *f* based on its connectivity within the GGM and the tentative matches of neighboring features to entries in the LMSD (Figure 2). Given the well-established class-specific retention behavior of lipids in RP-chromatography,^40^ predictions were restricted to neighboring features eluting within a defined rt window of *f*. For the AddNeuroMed data sets a window of ± 50 s was applied, while a broader window of ± 150 s was used for the metformin-HIIE data set to accommodate the assay’s longer chromatographic run time. These rt windows represent a practical compromise: wide enough to include a useful number of neighboring features of the same lipid class, yet narrow enough to limit false positives that could adversely affect scoring. The lipid class scoring framework involves three steps: evaluation of database class matches for (i) the feature of interest *f*, (ii) its direct neighbors (1-hop), and (iii) features connected via two-step paths (2-hop).

#### 1-Hop Score

Let *N*_1_(*f*) be the set of 1-hop neighbors of *f* in the GGM, and let ρ_*f*,*i*_ denote the partial correlation coefficient between features *f* and *i*. The score for subclass *c* ∈*C*, where *C* = {*c*_1_, *c*_2_, ..., *c*_*n*_} is the set of lipid subclasses, is then calculated by considering the 1-hop neighbors (the ego graph of *f*) with accurate mass matches. Here, **1**_{match(*i*)=*c*}_ is an indicator function which evaluates to one if feature *f*_*i*_ has a tentative match to a structure of subclass *c*, else it evaluates to zero.

$$S_{1}\left(c,f\right)=\underset{i\in N_{1}\left(f\right)}{\sum }1_{\left\{\mathrm{match}\left(i\right)=c\right\}}\left|\rho_{f,i}\right|$$

#### 2-Hop Score

Let *N*_2_(*f*) be the set of features connected to feature *f* by two-step paths in the GGM. For each 2-hop neighbor *k* ∈*N*_2_(*f*), the paths from *f* → *i* → *j* with the maximum product of absolute partial correlation coefficients *w*_*f*,*j*_ is considered:

$$w_{f,j}=\underset{i\in N_{1}\left(f\right)}{\max}\left|\rho_{f,i}\right|\times \left|\rho_{i,j}\right|$$

The 2-hop score for subclass *c* is then obtained by summing these path weights over all 2-hop neighbors whose tentative matches belong to subclass *c*:

$$S_{2}\left(c,f\right)=\underset{j\in N_{2}\left(f\right)}{\sum }1_{\left\{\mathrm{match}\left(j\right)=c\right\}}w_{f,j}$$

This weighting scheme downweighs contributions from 2-hop neighbors by taking the product of two partial correlation coefficients to reflect their more indirect association with the feature of interest.

#### Self-Weight Score

Since a feature *f* does not have a partial correlation with itself, its own class match is incorporated using the maximum absolute partial correlation coefficient among its 1-hop neighbors with the same class match. Let

$$M_{c}\left(f\right)=\underset{i\in N_{1}\left(f\right)}{\max}\left\{\left|\rho_{f,i}\right|:\mathrm{match}\left(i\right)=c\right\}$$

Denote this maximum value for subclass *c*. The self-weight score is then given by

$$S_{\mathrm{self}}\left(c,f\right)=\alpha \times M_{c}\left(f\right)$$

where α is a user-defined parameter representing the self-importance, set to 5 for all assays.

#### Final Subclass Score

The overall subclass score for feature *f* and subclass *c* combines the contributions from 1-hop neighbors, 2-hop neighbors, and the self-weighted score:

$$S\left(c,f\right)=S_{1}\left(c,f\right)+S_{2}\left(c,f\right)+S_{\mathrm{self}}\left(c,f\right)$$

The predicted subclass is taken as the top-ranking score, and the main class prediction is derived from the LMSD ontology.

### Quality Scores

To support the robust downstream interpretation of LMSD lipid class predictions, we developed three quality scores to quantify the reliability of these assignments. Due to the encoding of lipid class information in the GGM, neighboring features are expected to belong to the same lipid class. Therefore, when a features neighbors exhibit diverse lipid subclass assignments the quality of the prediction is expected to be lower. To quantify this, we computed a local Simpson’s index (LSI) for each feature *f*:

$$L\left(f\right)=\underset{c_{i}\in C}{\sum }P_{f}{\left(c_{i}\right)}^{2}$$

where *C* = {*c*_1_, *c*_2_, ..., *c*_*n*_} represents the set of lipid subclasses, and *P*_*f*_(*c*_*i*_) is the proportion of *f*’s neighbors predicted to belong to subclass *c*_*i*_. Due to differences in feature edge degree within the GGM, neighbors were defined as the *k* = 5 nearest neighbors in a uniform manifold approximation and projection (UMAP) embedding of the GGM min-max scaled, absolute weighted adjacency matrix (number of components = 2, cosine distance). LSI scores were subsequently min-max scaled across all features to bound the quality score between 0 and 1. Lower LSI values indicate that the lipid subclass predictions for a features nearest-neighbors are diverse.

We also computed a partial correlation based (PCOR) quality score for each feature *f* using the GLC subclass prediction scores. Specifically, given the top-ranked subclass prediction score *S*(*ĉ*, *f*), the score was normalized by the sum of the top *K* = 10 subclass scores:

$$Q\left(f\right)=\frac{S\left(ĉ,f\right)}{\overset{K}{\underset{i=1}{\sum }}S\left(c_{i},f\right)}$$

To facilitate comparison across all features in the data set, PCOR scores were transformed using a uniform quantile transform so that higher values reflect stronger support from database matches and partial correlation coefficients. Finally, the product score was defined as the product of the LSI and PCOR scores and subsequently transformed with a uniform quantile transform across the data set, integrating both local neighborhood consistency and strength of lipid class supporting partial correlation coefficients.

### The Closest-*m*/*z* Model

As a baseline model against which to compare GLC, we defined the closest-*m*/*z* model based on conventional accurate mass searching against the LMSD within a ten-ppm tolerance. For the AddNeuroMed-LPOS/LNEG assays, observed *m*/*z* values were matched to all relevant ion forms listed for the corresponding ion mode in LIPID MAPS ‘bulk structure searches’ service (listed in section S.1.6). For the metformin-HIIE data set, which contains feature-grouped data, estimated neutral masses were matched against monoisotopic masses in the LMSD. The lipid class of the closest ppm match was assigned as the predicted class. Ties arise when multiple structures share the same molecular formula but belong to different lipid classes. For the calculation of classification metrics, only one lipid class can be top-ranking. To evaluate the average performance, ties were resolved by random sampling, and the metrics were computed across 1,000 random seeds. The mean and standard error of the mean for the classification metrics were reported.

### UMAP Visualization of GGMs and Statistical Analysis

Because of the large number of features, a standard graph layout algorithm was not employed. Instead, the network structure was visualized by applying UMAP to the min-max scaled, absolute-weighted adjacency matrix of the GGM. UMAP was configured to reduce the data to two components and parametrized with the cosine distance. To emphasize global relationships in the lipid class, the minimum distance parameter was set to 1, and the number of neighbors was set to 2% of the total number of assay features.

The methodology outlined by Chen et al.^41^ was used to test whether lipids belonging to the same class are more closely grouped in the UMAP embedding of the GGM than would be expected by chance. Specifically, the test follows a binomial framework in which the probability that a lipid’s nearest neighbor belongs to the same class is modeled under a binomial null distribution. A one-tailed Z-test is then used to evaluate whether lipid class annotations occur as one–nearest neighbors (1-NN) in the UMAP embedding more frequently than expected by chance. This test was applied to lipid class annotations at both the lipid subclass and main class levels derived from targeted feature extraction, as well as to lipid class predictions from the GLC model. As applied to lipid subclass, let μ_0_ be the proportion of all ground-truth annotations from targeted feature extraction belonging to that subclass, μ be the proportion of annotations in that subclass whose 1-NN also belongs to the same subclass, and σ be the standard error, computed as

$$\sigma =\sqrt{\frac{\mu_{0}\left(1-\mu_{0}\right)}{n}}$$

where *n* is the number of annotations in the subclass. The Z-score is then calculated as

$$z=\frac{\mu -\mu_{0}}{\sigma }$$

and quantifies how much more frequently members of a given subclass are nearest neighbors to each other than would be expected under the null model defined by their overall frequency.

### Lipid Class-Level Enrichment Analysis

Class enrichment analysis was performed to test whether features associated with a given lipid subclass showed a collective difference between sample groups in the AddNeuroMed-LPOS/-LNEG data sets. Analyses were conducted separately using either GLC-predicted subclass predictions or ground-truth annotations from targeted feature extraction, and the results were not combined across ionization modes.

Each feature was modeled using logistic regression with phenotype as the outcome, adjusting for age and sex as covariates. For each feature, the regression coefficient was tested, and the corresponding *p*-value was extracted. Subclass-level significance was assessed using an empirical Fisher’s method. For a given subclass *c*, the observed test statistic was calculated as

$$T_{c}^{\mathrm{obs}}=-2\underset{i\in c}{\sum }\ln\left(p_{i}\right)$$

where *p*_*i*_ is the feature-level *p*-value from logistic regression. To account for correlation among features, especially due to multiple ion forms of the same analyte, we generated an empirical null distribution by permuting phenotype labels 10,000 times. For each permutation *b*, the test statistic was recalculated as

$$T_{c}^{\left(b\right)}=-2\underset{i\in c}{\sum }\ln\left(p_{i}^{\left(b\right)}\right)$$

where *p*_*i*_^(*b*)^ is the permuted *p*-value for feature *i*. The empirical *p*-value for subclass *c* was then obtained as

$$p_{c}=\frac{1+\overset{B}{\underset{b=1}{\sum }}1\left(T_{c}^{\left(b\right)}\geq T_{c}^{\mathrm{obs}}\right)}{1+B}$$

Multiple testing correction was applied using the Benjamini–Hochberg false discovery rate procedure (α ≤ 0.05). Testing was restricted to subclasses with sufficient representation: ≥15 features for GLC predictions and ≥3 features for targeted annotations, reflecting the lower coverage of annotations. As mapping ground-truth annotations from targeted feature extraction to the feature table was unnecessary, we used the entire set of annotations. This comprised 287 lipid subclass annotations and 4,693 features for AddNeuroMed-LPOS, and 99 subclass-specific annotations with 1,865 features for AddNeuroMed-LNEG.

To quantify concordance in subclass ranking, we calculated a cumulative rolling Jaccard similarity. Comparisons were restricted to only subclasses detected in both analyses. Four sets of comparisons were made: (1) GLC predictions versus targeted annotations in positive ion mode, (2) GLC predictions versus targeted annotations in negative ion mode, (3) GLC predictions between positive and negative ion modes, and (4) targeted annotations between positive and negative ion modes. Given two ranked lists, *R*_1_ and *R*_2_, the cumulative rolling Jaccard at rank *k* was calculated as

$$J_{k}=\frac{\left|\left\{R_{1}\left[1:k\right]\right\}\cap \left\{R_{2}\left[1:k\right]\right\}\right|}{\left|\left\{R_{1}\left[1:k\right]\right\}\cup \left\{R_{2}\left[1:k\right]\right\}\right|}$$

The area under the rolling Jaccard curve (AUC) was computed using the trapezoidal rule and normalized to the interval [0,1] by dividing by the maximum possible area:

$${\mathrm{AUC}}_{\mathrm{norm}}=\frac{\overset{n-1}{\underset{k=1}{\sum }}\frac{J_{k}+J_{k+1}}{2}}{n-1}$$

where *n* is the length of the ranking. Statistical significance was assessed using 1,000 permutations to generate a null AUC distribution, AUC_norm_^(*b*)^, and the empirical *p*-value was calculated as

$$p_{\mathrm{emp}}=\frac{1+\overset{B}{\underset{b=1}{\sum }}1\left({\mathrm{AUC}}_{\mathrm{norm}}^{\left(b\right)}\geq {\mathrm{AUC}}_{\mathrm{norm}}^{\mathrm{obs}}\right)}{1+B},B=1000$$

## Results

### Lipid Class Structure in Gaussian Graphical Models

GLC predicts lipid class by leveraging accurate mass searching in combination with GGM structure inferred from feature intensities. While previous studies have shown that GGM organization can encode lipid class in lipidomic and metabolomic data sets, these prior observations do not fully represent the challenges of estimating a GGM from a full untargeted feature table.^21,24^ In untargeted data sets, multiple features often correspond to the same analyte, and the number of features generally exceeds the number of samples,^38^ requiring the use of regularization techniques for GGM estimation. As a result, the capacity of GGMs to encode lipid class may be less pronounced for untargeted data sets.

To evaluate the encoding of lipid class information in untargeted LC-MS data sets, we examined the estimated GGM for the AddNeuroMed-LPOS data set (600 patients, 4849 features in the main subgraph of the GGM; Figure 1). Given the large number of features, UMAP was applied to the weighted adjacency matrix to facilitate network visualization. Figure 1A,B, shows that feature rt and *m*/*z* vary smoothly across the GGM, consistent with expectations that lipids exhibit class-specific retention time behavior and class members will often tend to be separated through few reaction steps, often resulting in low *m*/*z* differences. We then examined the GGM structure for the 183 features with ground-truth lipid class annotations to determine whether nearby features shared the same lipid class. As shown in Figure 1C,D, annotations that are closely positioned in the embedding space frequently share the same LMSD main and subclass. This was confirmed with statistical testing, with all lipid classes containing more than four annotations exhibiting significant clustering in the embedding space (adjusted P-value of <0.05; Tables S4 and 5). Comparable results were obtained for the AddNeuroMed-LNEG and metformin-HIIE data sets (Tables S6–9), indicating that the encoding of lipid class information in GGM structure is robust across data sets and ionization modes.

**Figure 1:**
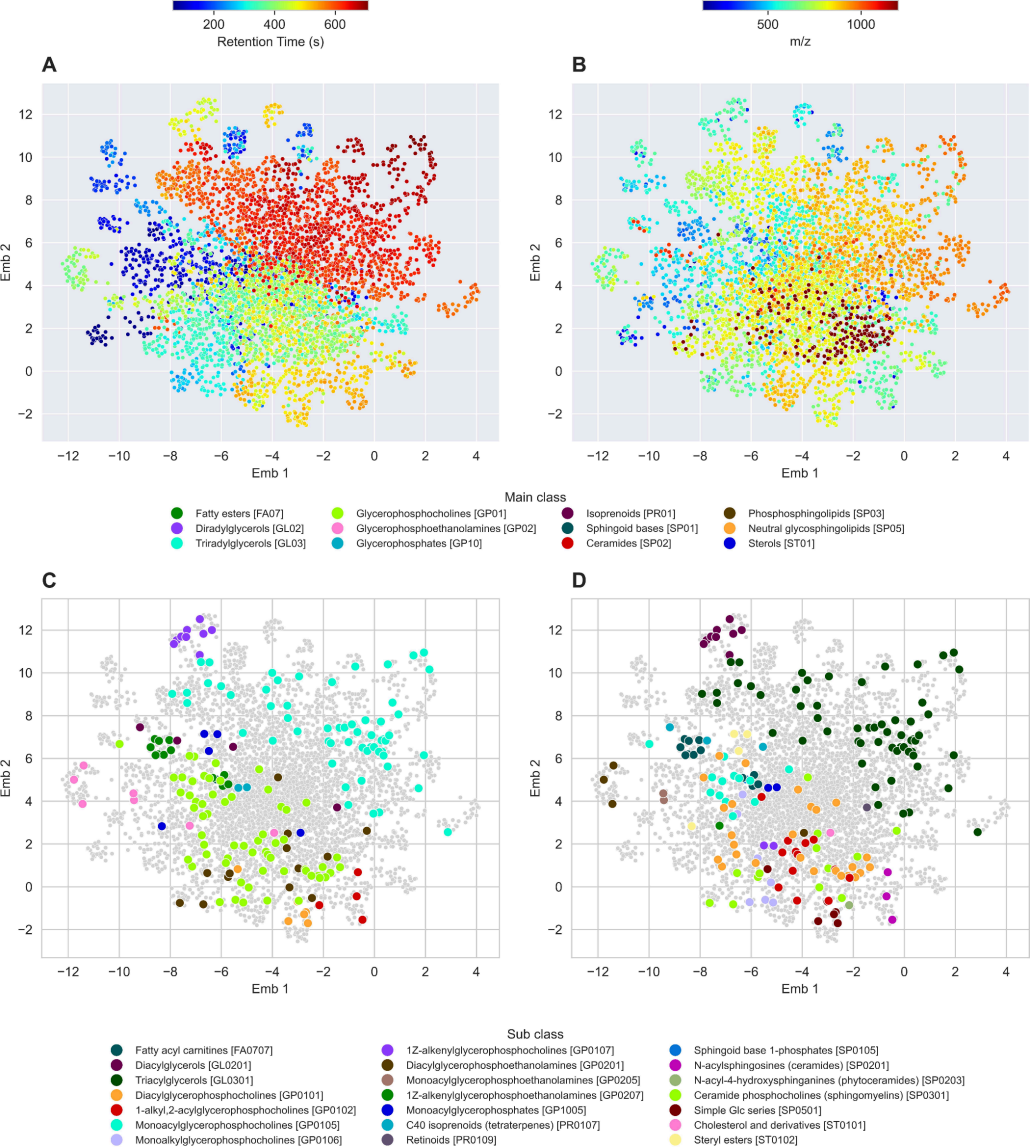
Two-dimensional UMAP embedding of the estimated Gaussian graphical model (GGM) constructed from feature intensities in the AddNeuroMed-LPOS data set. Each point represents a detected feature. (A) Features colored by rt (seconds). (B) Features colored by *m*/*z*. (C) LMSD main lipid class ground-truth annotations from targeted feature extraction and (D) LMSD lipid subclass annotations in the GGM.

### GgmLipidClassifier

GLC predicts lipid classes by integrating the feature-intensity-estimated GGM with database matching against the LMSD (Figure 2A). The approach follows a guilt-by-association rationale: rather than relying on a single *m*/*z* database search, GLC leverages the local structure of the GGM to reduce the assignment uncertainty. As inputs, GLC requires a feature table and an LMSD. To score a feature, its immediate neighbors in the GGM (ego graph) are identified, and their observed *m*/*z* values are searched against the LMSD. Subclass scores are then calculated from the absolute partial correlation coefficients of neighbors with matching database entries. This procedure is then extended to include two-hop neighbors and the features themselves.

**Figure 2:**
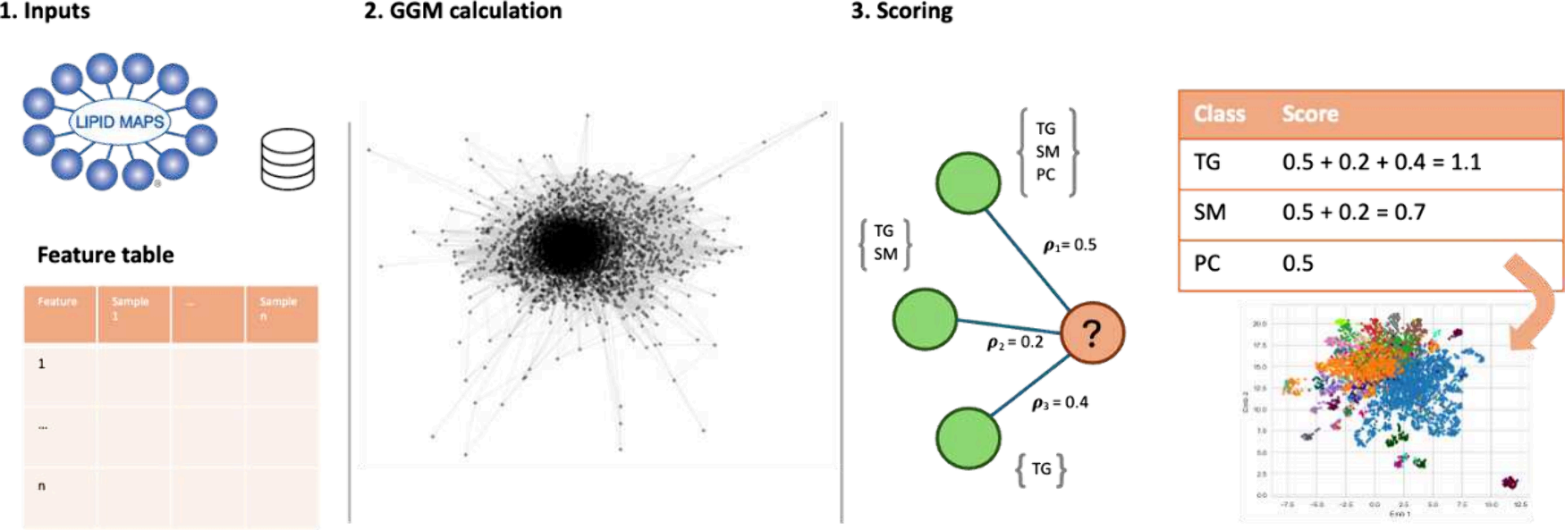
Overview of GggmLipidClassifier (GLC). GLC requires the LIPID MAPS structural database and a feature table as inputs. Using feature intensities, a Gaussian Graphical Model (GGM) is estimated. For a given query feature (shown in orange), each lipid subclass score is calculated as the sum of the absolute partial correlation coefficients of its one-hop neighboring features that have a database match to the subclass. Information from two-hop neighboring features and the feature of interest is then considered (not shown). The subclass with the highest overall score is assigned as the prediction lipid class for a feature.

To evaluate GLC, we first constructed naïve baseline models to assess the effect of incorporating GGM neighboring features in class prediction. The baseline closest-*m*/*z* match model predicts class based on the accurate mass search of the feature of interest. Correct predictions using this approach depend on both database coverage and whether the correct ion form was included. Ties among top-ranking class predictions were common, with 30.1% (55 out of 183) annotations in the AddNeuroMed-LPOS data set corresponding to multiple lipid subclasses sharing the same molecular formula. To account for this isomer distribution and enable the calculation of classification metrics, ties were resolved by random sampling. Figure 3A shows an instance (random seed zero) of the closest-*m*/*z* model for the 183 annotations in the AddNeuroMed-LPOS data set, while Figure 3B presents the corresponding GLC predictions, with overall performance summarized in Figure 3C. Unlike the closest-*m*/*z* baseline, GLC produced a single top-ranking prediction for each feature, avoiding subclass ties. Furthermore, GLC achieved a more balanced performance across subclasses, with a macro F1 score of 0.70, whereas the baseline model was skewed toward subclasses such as triacylglycerols and ceramide phosphocholines, achieving an average macro F1 score of 0.33. Overall, GLC outperformed the baseline model, achieving 90% accuracy at the main class-level and 86% at the subclass level, compared to 52% (subclass) and 62% (main class) for the closest-*m*/*z* model. Furthermore, GLC performance was consistent across additional data sets. In the AddNeuroMed-LNEG data set (ESI(−)), accuracy was 82% for the main class and 72% for the subclass (Figure 3D), while in the metformin-HIIE data set, acquired on a different platform with different preprocessing workflows, GLC achieved 83% accuracy at both class levels (Figure 3E). The contribution of GGM neighboring features to class scores was further evaluated using a permuted baseline, in which the feature-to-node mappings were randomized. This led to a substantial decline in performance, with macro F1 scores of 0.07 and 0.03 on the AddNeuroMed-LPOS data sets for lipid main class and subclass, respectively (Figure S4). As GGM estimation from limited data is susceptible to random noise, we assessed the influence of sample size on GLC performance using the ground-truth annotations (Figure S5). Increasing the number of samples improved lipid class prediction and reduced variability across random subsamples, with performance gains plateauing around 100 samples. Collectively, these results indicate that GLC substantially improves prediction accuracy over baseline models by leveraging GGM structure and produces a more balanced and robust lipid class assignment across data sets.

**Figure 3:**
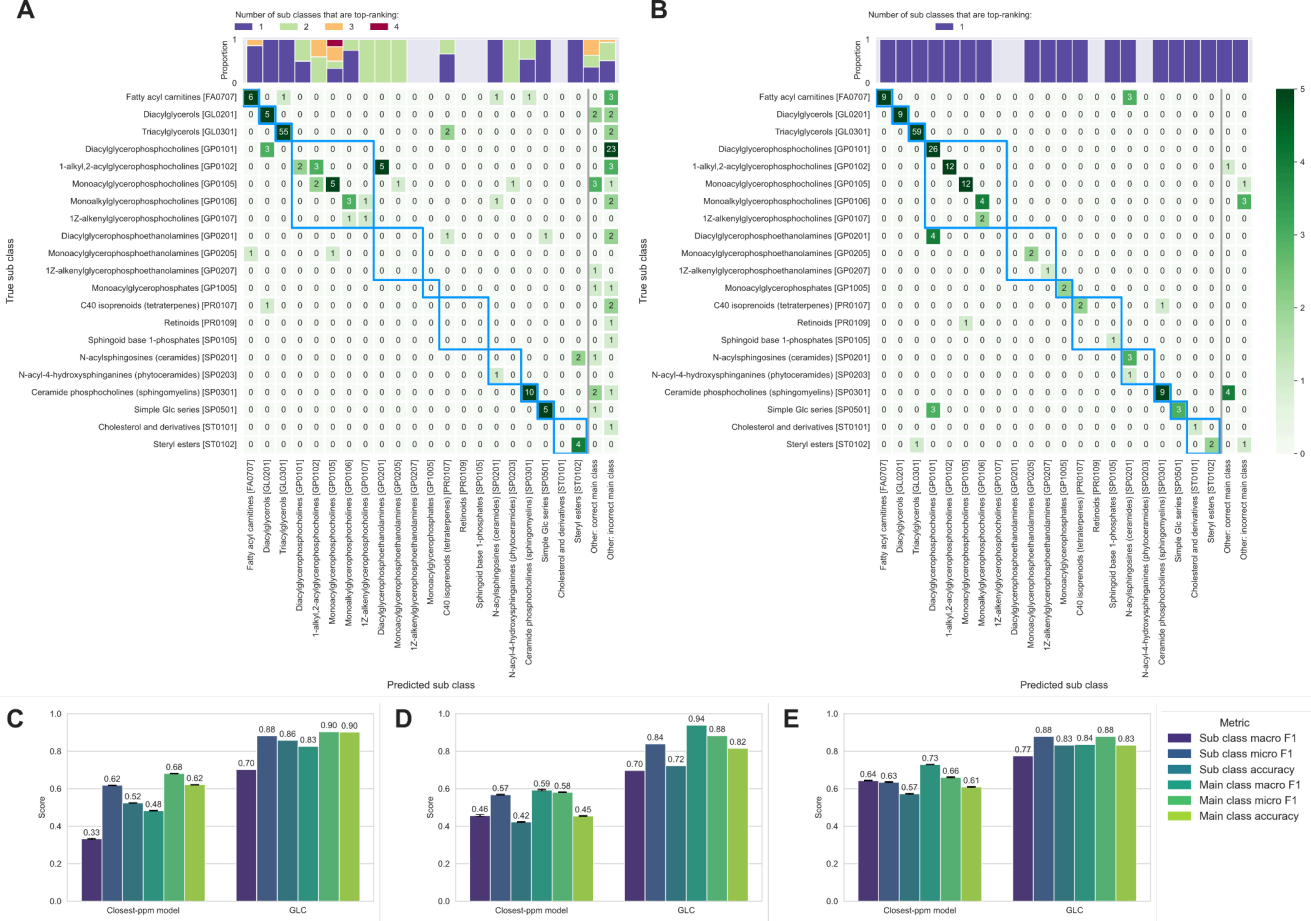
Hierarchical confusion matrices based on the LIPID MAPS structural database (LMSD), comparing lipid class predictions from (A) the closest-*m*/*z* model and (B) GgmLipidClassifier against ground-truth annotations for the AddNeuroMed-LPOS data set. Blue bounding boxes indicate lipid subclasses belonging to the same main class. Lipid subclass predictions absent from the annotations are grouped into the columns ‘Other: correct main class’ and ‘Other: incorrect main class’. The classification metrics, i.e., macro F1, micro F1, and overall accuracy, for main- and subclass-level predictions for the (C) AddNeuroMed-LPOS, (D) AddNeuroMed-LNEG, and (E) metformin-HIIE data sets. Error bars represent the standard error of the mean across 100 random-seed runs, shown only for the closest-*m*/*z* model, as GgmLipidClassifier yields identical results across runs.

GLC computed lipid class predictions for all features within the main GGM subgraph, covering 99.81% of features in the AddNeuroMed-LPOS data set, 99.62% in AddNeuroMed-LNEG data set, and 93.08% in metformin-HIIE. This coverage contrasts with many annotation tools, which are often limited by the scope of seed annotations or by reliance on phenotype-associated effect sizes. Lipid class GLC predictions for subclasses with more than 15 members in AddNeuroMed-LPOS are visualized in the UMAP embedding of the GGM structure (Figure 4A), where predictions from the same subclass tended to cluster and aligned with the distribution of the lipid subclass of ground-truth annotations (Figure 1D). In addition, 27 out of 28 lipid subclasses showed statistically significant nearest-neighbor UMAP clustering (adjusted P-value <0.05; Table S10). Consistent with the GGM structure encoding lipid class, GLC predictions were less locally diverse than those of the closest-*m*/*z* baseline (evaluated using the local Simpson’s index on the 65.85% of features for which the baseline produced predictions; Figure S7). We also compared the distribution of subclass predictions in the rt–*m*/*z* domain with the in-house database for the AddNeuroMed-LPOS data set, and the results visually confirm a general concordance (most frequent classes Figure 4B, all Figure S6).

**Figure 4:**
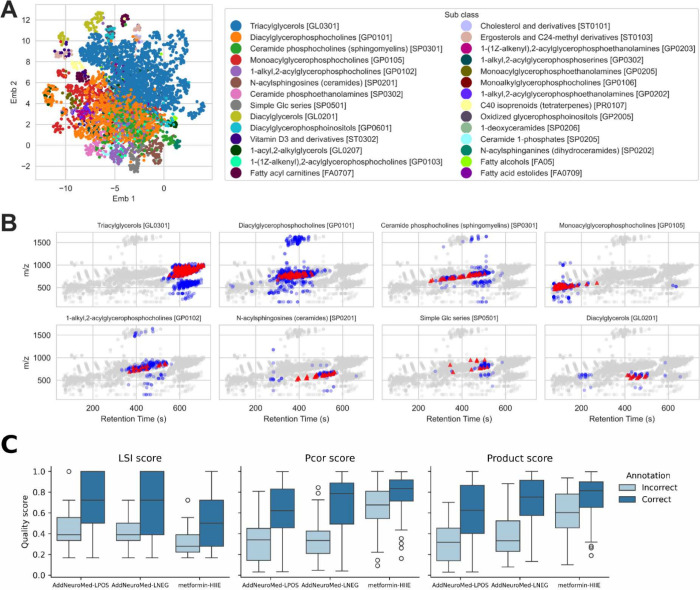
(A) Two-dimensional UMAP embedding of the estimated Gaussian Graphical Model for the AddNeuroMed-LPOS data set. Each point is colored according to the leading subclass prediction from GgmLipidClassifier (GLC). (B) *m*/*z*–rt plots show all detected features in gray, GLC-predicted lipids in blue, and lipids present in the in-house database in red. (C) Quality scores for GLC predictions with ground-truth annotations are stratified by whether the Lipid Maps subclass was correctly predicted. Scores include the Local Simpson’s Index (LSI), the partial correlation score (PCOR), and the product of LSI and PCOR.

To facilitate downstream analysis and prioritize the search space for structural elucidation, we developed quality scores for the lipid class predictions. The first metric is the local Simpson’s index (LSI), based on the principle that neighboring features in the GGM should share the same lipid class, and a partial correlation-based score (PCOR), which evaluates evidence from database matches and correlation strength relative to lower-ranking class predictions. A quantile-transformed product of the scores was also introduced to emphasize predictions supported by both metrics. Across three data sets, correct predictions had consistently higher scores on ground-truth annotations (Figure 4C), with all differences statistically significant (adjusted *p*-value <0.05, Table S11). However, interpretation of the scores is data set-specific and not transferable. For instance, the metformin-HIIE data set showed distinct distributions compared to the AddNeuroMed data sets, likely due to its grouped feature table; nearest neighbors are from different analytes, lowering the LSI score, and fewer database matches may limit the ability of the PCOR score to stratify true from false predictions.

### Lipid Class Enrichment Analysis

Lipidomics aims to comprehensively characterize the lipid composition of a biological system, but its scope is often constrained by the limited number of annotations.^8^ GLC provides class-level predictions for most detected features, enabling the analysis of class-level patterns that might otherwise be overlooked when relying on a small subset of annotated lipids. As a case study, we investigated lipid class patterns in the AddNeuroMed-LPOS/LNEG data sets, comparing serum class levels between Alzheimer’s disease (AD) and cognitively healthy controls (CTL). Log 2-fold changes for all GLC predictions grouped by Lipid Maps main class are shown in Figure 5A for the AddNeuroMed-LPOS data set. To coarsely reflect lipid structure within a lipid class, predictions are ordered in ascending elution order. Within a lipid class, local block structures from features exhibiting similar fold-change trends were often observed, supporting the collective association of class feature groups with AD. Notably, 87.78% of the LMSD main class predicted triradylglycerol features showed a decreased FC, indicating lower levels in the AD serum.

**Figure 5:**
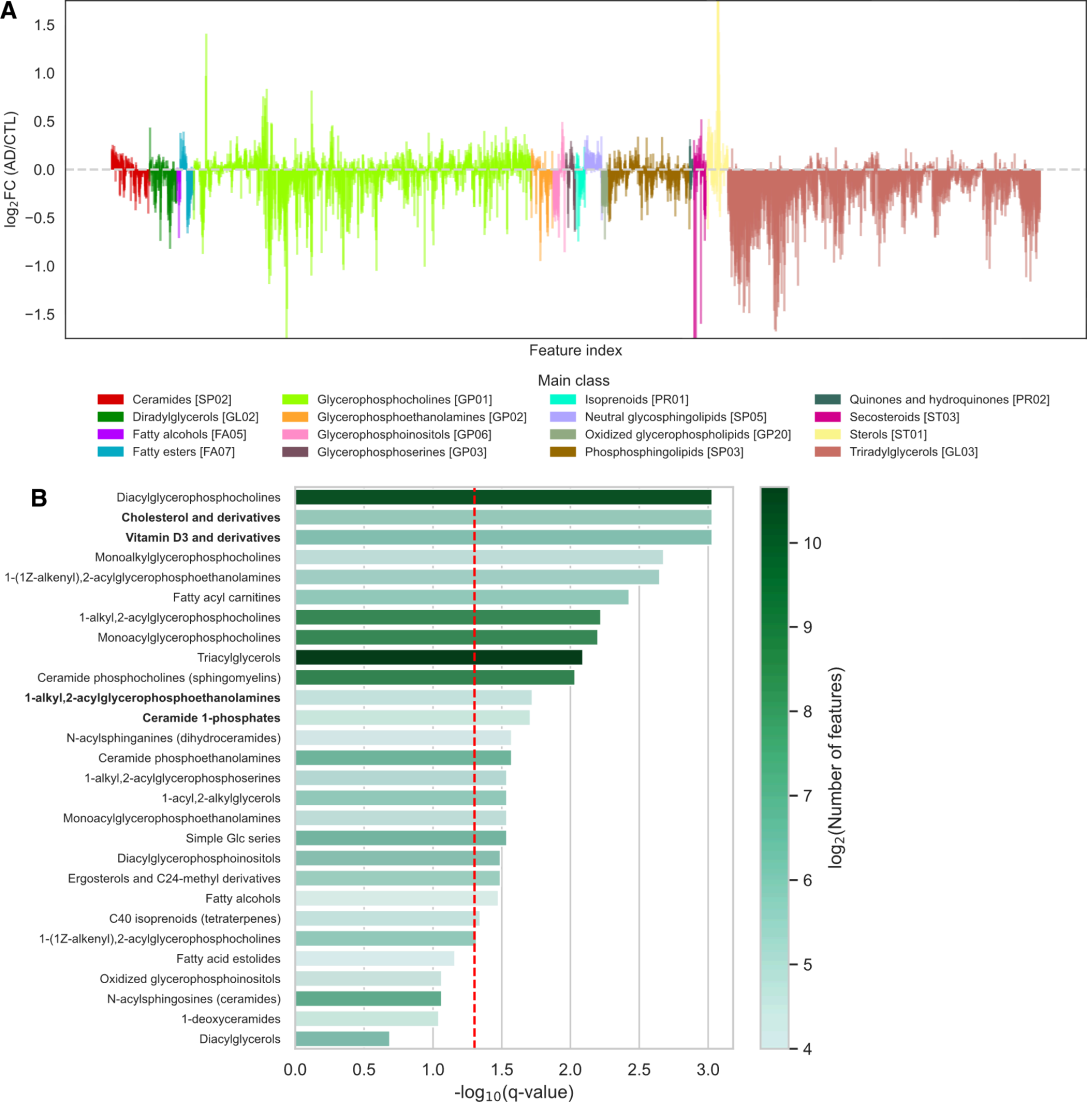
Class-level analysis of GgmLipidClassifier predictions in the AddNeuroMed-LPOS data set. Only classes with ≥ 15 predictions are shown. (A) Predictions are grouped by main lipid class and ordered by ascending elution time. The log-2-fold change (FC) of median relative abundance in Alzheimer’s disease (AD) versus cognitively healthy controls (CTL) is displayed. (B) Lipid Maps subclass enrichment assessed using an empirical Fisher’s method. The red line indicates a *p*-value of 0.05. The color bar represents log-two number of predicted features.

To compare chemical classes between the AD and CTL groups, we performed a chemical enrichment analysis. A widely used method is ChemRICH,^42^ which assesses whether metabolites within a class collectively differ between groups by testing the *p*-value distribution against a uniform null using the Kolmogorov–Smirnov method. However, this assumption is problematic for GLC predictions, as there are many highly correlated features, including multiple ion forms from the same analyte, which can inflate type I errors. To address this, we employed phenotype permutation to generate an empirical null distribution that preserves the correlation structure among metabolites. Specifically, each metabolite was modeled using logistic regression with AD versus CTL as the outcome and adjusted for the covariates age and sex. *p*-values were extracted for each feature, and class-level significance was assessed using an empirical Fisher’s method, which tests whether the observed sum of associations within a class (the sum of negative log-transformed *p*-values) could be obtained under the null distribution from phenotype permutation. We applied this analysis to GLC-predicted classes (≥15 features) and ground-truth class annotations from targeted feature extraction (≥3 annotations) in both ionization modes of the AddNeuroMed study (Figure 5B, Figure S8). The ranked significance of lipid classes was generally consistent between GLC predictions and annotations and across ion modes (Figure S9). Concordance was quantified using the rolling Jaccard method, which measures cumulative overlap in ranked lists. The area under the similarity curves was high, indicating strong agreement between GLC predictions and corresponding class annotations (Figure S10 within the same mode, and this overlap was statistically significant compared to random rankings (*p* < 0.05). Ground-truth annotations also showed complete ranking overlap across ion modes, though limited to four common subclasses. Ranking overlap between GLC prediction across modes was not significant (*p* = 0.1439). However, all 14 shared subclasses consistently agreed on whether the lipid class was significant or not (Figure 5B, Figure S8).

Using GLC predictions, class-level information was available for a larger proportion of the data sets, compared to manual annotations. In the AddNeuroMed data sets, this enabled class enrichment analyses for 16 and 21 lipid classes that were not represented in the annotations in positive and negative ionization modes, respectively. Although our ability to infer AD etiology is limited by the retrospective study design and available covariates, the expanded coverage from GLC predictions provided biological insights not accessible from ground truth annotations and could be used to guide downstream analysis. For example, the LMSD subclass ‘cholesterol and derivatives’ was not accessible from ground-truth annotations but was accessible from GLC predictions in ESI­(+), and the LMSD subclass was significantly associated with AD (Figure 5B). Cholesterol is implicated in AD pathobiology as a regulator of β-amyloid generation and clearance,^43^ and meta-analyses report elevated serum cholesterol levels in AD patients.^44^ Similarly, the ‘Vitamin D3 and derivatives’ class was only captured by GLC predictions and was significant in both ion modes. Meta-analysis has reported consistently reduced serum 25-hydroxyvitamin D levels in AD,^45^ and vitamin D-related metabolites and pathways are linked to amyloid pathology.^46^ Another LMSD subclass, 1-alkyl,2-acylglycerophosphoethanolamines (also referred to as plasmalogen glycerophosphoethanolamines), was not sufficiently represented in annotations for class analysis but was significantly associated with AD in GLC predictions across both ion modes. Plasmalogen glycerophosphoethanolamines are well-established as relevant to AD, with levels correlating to cognitive deficits and disease severity.^47^ Finally, ceramide 1-phosphates were only captured in AddNeurMode-LPOS and were significantly associated with AD; total levels of these lipids have recently been reported to be elevated in post-mortem AD brain tissue.^48^ Overall, GLC is consistent with class information available from ground-truth annotations, while its expanded coverage reveals additional biologically plausible class-level associations.

## Discussion

GLC systematically predicts lipid classes for features detected in untargeted LC-MS lipidomics by combining traditional database matching of observed *m*/*z* values with GGMs estimated from feature intensity, where network structure has been shown to associate with lipid class.^21,24^ GLC unifies these components with a systematic framework that requires only a feature table as the user input (no annotations or MS2). Across three human serum and plasma data sets, GLC achieved 72–86% accuracy at the LMSD subclass level and 82–90% at the main class level, outperforming the baseline closest-*m*/*z* model, which showed lower accuracy (42–57% subclass; 45–62% main class). By incorporating the GGM network structure, GLC reduces the ambiguity associated with multiple potential matches between features and lipid classes from accurate mass searching. Accordingly, GLC produced fewer top-ranking ties between lipid classes, with only 8 tied features compared to 580 in the closest-*m*/*z* model for the AddNeuroMed-LPOS data set. Furthermore, by leveraging the database matches of neighboring features in the GGM, GLC is positioned to generate class predictions for features that conventional database searches might miss because of limited curation.

GLC is not intended to replace MS2-based lipid-class prediction. Fragmentation spectra offer additional structural information beyond the *m*/*z* of a feature, demonstrate greater cross-platform generalizability, and support large-scale machine learning.^10^ For example, MS2Lipid achieves an average LMSD subclass prediction accuracy exceeding 87.2% in cross-instrument validation data sets.^11^ However, data-dependent acquisition of MS2 spectra is biased toward the most abundant ions. While estimates in the literature vary and strongly depend on sample, platform, and protocol, MS2 spectra are typically not acquired for most detected features.^8,9^ Additionally, samples are often processed in full-scan mode, and MS2 is collected afterward on pooled quality controls or MS2 is collected for only targeted features. Given that untargeted lipidomics aims to comprehensively characterize the lipid profile of a biological system, GLC can be a valuable addition to a lipidomic workflow by enabling class prediction for the majority of the features. Ultimately, when incorporating lipid-class prediction software into a workflow, the software choice requires balancing predictive power with coverage. Scenarios where GLC may be particularly advantageous include those with limited MS2 acquisition, when there are difficulties matching MS2 spectra to features, and investigations of low-abundance features. Furthermore, network propagation of information between features is a well-established approach that has proven to be useful for enhancing interpretation.^49^ Future research would benefit from continued investigation into how to jointly analyze MS1 and MS2 information for lipid class prediction, particularly by considering GGM structure, which has the potential to improve both coverage and predictive accuracy.

An interesting lipid subclass predicted for 16 features in the AddNeuroMed-LPOS data set was fatty acid estolides. Although these compounds are poorly characterized in humans, they could plausibly originate from endogenous synthesis^50^ or xenobiotic exposure. Alternatively, these signals may have arisen from the in-source fragmentation of triacylglycerides (TGs). The resulting diacylglycerol-like fragments, produced through the loss of a fatty acyl chain, share the same nominal mass as fatty acid estolides and can therefore be misclassified by accurate-mass database approaches, such as GLC. Supporting this interpretation, most fatty acid estolide predictions eluted at retention times consistent with TG, 75% of the features had TG as the second-highest-ranking prediction, and the class exhibited a low average PCOR quality score (0.098), indicating weak database support within the local GGM structure. Overall, these findings highlight that GLC predictions should be interpreted with an understanding of mass spectrometry and a consideration of the associated GLC quality scores.

Feature annotation is an essential step in untargeted lipidomics workflows but tends to be time- and labor-intensive. As GLC requires only a feature table and does not depend on prior annotations, it can be applied immediately after preprocessing, providing lipid-class predictions along with associated quality scores. These early insights can support the annotation process by offering starting points for structural elucidation and helping to prioritize which analytical standards should be purchased. Furthermore, GLC predictions can be used to investigate the lipid classes associated with biological outcomes. We found that lipid class enrichment analysis based on GLC predictions was highly consistent with results obtained from ground-truth annotations for identifying altered lipid classes between Alzheimer’s disease and cognitively healthy controls. In addition, the broader coverage of GLC allowed the identification of lipid subclasses not represented in the ground-truth data, including cholesterol and derivatives, vitamin D3 and derivatives, plasmalogen glycerophosphoethanolamines, and ceramide 1-phosphates, which were found to be altered.

There are several directions for future work that could improve the performance and scope of the GLC. Instead of analyzing different ion modes acquired on the same chromatography and instrument separately, they could be integrated, which may provide additional information useful for lipid class prediction. Additionally, there is a need for approaches that perform well for data sets with a small sample size. Sample size is important for robust GGM estimation, and our sensitivity analysis on the AddNeuroMed-LPOS data set suggests that about 100 samples are needed for performance gains to plateau. One interesting direction that could help address this is the integration of prior knowledge into GGM estimation.^51^ In this context, prior knowledge could be defined using information that is broadly available across features, such as mass differences corresponding to known biochemical transformations, which could help regularize the network and improve estimation in low-sample settings. Another important consideration is the completeness and suitability of the LMSD; while it is a comprehensive resource, it is not organism-specific and does not encompass all lipids expected within a biological system, which may have adverse effects on GLC accuracy, particularly for poorly curated lipid classes. Finally, while we have applied GLC to lipidomics data sets, the methodology could easily be adapted and generalized to (nonlipid) metabolomic data sets, and future work should aim to validate GLC performance for these data sets.

In conclusion, GLC predicts lipid classes by integrating the relationships between features in a GGM with accurate mass-based database matching. The method requires no prior annotations or training stage and can assign lipid classes to most features in a data set. While the average classification accuracy (78–90% for LMSD main class and 70–86% for subclass) is lower than MS2-based approaches, GLC provides substantially broader coverage, potentially offering a more comprehensive view of the lipidome. As it requires only a feature table as input, GLC can be implemented early in the lipidomics workflow to support structural elucidation endeavors. Additionally, its predictions can be incorporated into lipid class enrichment analyses to uncover biologically relevant lipid patterns and facilitate early hypothesis generation. The GLC software is freely available as an open-source Python package.

## Supplementary Material



## Data Availability

The software developed as part of this study is available as an open-source Python package at: https://github.com/trix98/GLC. The AddNeuroMed-LPOS/-LNEG data sets are available at: https://zenodo.org/records/15701829. The metformin-HIIE data is available on Metabolomics Workbench with study ID: ST003807.

## Notes

Data set descriptions and targeted feature extraction workflows, additional methods on database matching using mass differences and retention time-logP filtering, permutation analysis of GGM contribution to prediction performance, lipid class clustering in GGM structure, sensitivity to sample size, retention time–*m*/*z* plots of lipid classes, quality score evaluation and comparison to closest-*m*/*z* model, lipid class enrichment analysis results, and comparison across ion modes (PDF)
