# Supplementary material for: Lipid Class Prediction from MS1 Data using Gaussian Graphical Models
Source: Anal Chem. 2026 May 20;98(21):15379–91. doi: 10.1021/acs.analchem.5c08067 (PMC13234823; doi:10.1021/acs.analchem.5c08067)
Supplement: Supplementary file 1 [file ac5c08067_si_001.pdf]

Supporting Information

Lipid Class Prediction from MS1 Data using Gaussian Graphical Models

Thomas Rix[1], Caroline Jane Sands[2], Alma Villaseñor[3, 5], Ana Gradillas[3], Coral Barbas[3], Elena Chekmeneva[2], Elizabeth J Want[4], and Timothy M D Ebbels[1]

[1]Section of Bioinformatics, Division of Systems Medicine, Department of Metabolism, Digestion and Reproduction, Faculty of Medicine, Imperial College London, London W12 0NN, U.K.
[2]National Phenome Centre, Department of Metabolism, Digestion and Reproduction, Imperial College London, London W12 0NN, U.K.
[3]Centro de Metabolómica y Bioanálisis (CEMBIO), Facultad de Farmacia, Universidad San Pablo-CEU, CEU Universities, Urbanización Montepríncipe, Boadilla del Monte, Madrid 28660, Spain
[4]Section of Bioanalytical Chemistry, Division of Systems Medicine, Department of Metabolism, Digestion and Reproduction, Faculty of Medicine, Imperial College London, London W12 0NN, U.K.
[5]Departamento de Ciencias Médicas Básicas, Instituto de Medicina Molecular Aplicada (IMMA) Nemesio Díez, Facultad de Medicina, Universidad San Pablo-CEU, CEU Universities, Boadilla del Monte 28668, Spain

April 22, 2026

Contents

1 Methods **S3**

1.1 AddNeuroMed study datasets . . . S3
1.2 Mapping Targeted Feature Extraction Results to the Feature Table . . . S3
1.3 Metformin high-intensity interval exercise (Metformin-HIIE) study dataset . . . S3
1.4 Estimation of neutral mass candidates for un-grouped feature tables . . . S4
1.5 LogP Filtering of Database Matches . . . S6
1.6 The Closest-m/z Model . . . S7

2 Supplementary Results **S7**

2.1 Graph Structure Null model . . . S7
2.2 Annotation class clustering in GGM structure . . . S8
2.3 Number of samples and GLC performance . . . S10
2.4 Subclass Prediction clustering in GGM structure . . . S12
2.5 RT-MZ plots AddNeuroMed-LPOS dataset . . . S13
2.6 Quality score statistical testing . . . S13
2.7 Lipid Subclass Enrichment Analysis . . . S15

1 Methods

1.1 AddNeuroMed study datasets

Variable	Category / Statistic	N (%)
**Disease Group**	AD	173 (28.22%)
	CTL	170 (27.73%)
	MCI	270 (44.05%)
**Sex**	Female	319 (52.04%)
	Male	294 (47.96%)
**Age (years)**	Median (IQR)	77 (73–81)

Table S1: AddNeuroMed cohort characteristics. Continuous variables (age) are presented as median with interquartile range (IQR). Categorical variables (disease group, sex) are presented as number of participants with percentages in parentheses. Alzheimer's Disease (AD), cognitively healthy control (CTL), mild cognitive impairment (MCI).

Targeted Feature Extraction:
The signals of the annotated lipids from the AddNeuroMed study have been extracted from the experimental MS1 data using an open-source R package, peakPantheR [1] which leverages the knowledge gained from the previously performed full annotation and identification with available standards of the detectable LC-MS signals in the reference biofluid samples used for each assay developed in the National Phenome Centre [2].
Metabolite annotation and identification was performed using a combination of experimental MS/MS fragmentation and computational tools. We used the Metabolomics Standards Initiative (MSI) criteria [3] to define the level of confidence in each metabolite's annotation. Metabolites with an MSI level of 1 were identified by matching signals to entries in an in-house database of reference chemical standards. We have assigned an MSI level of 2 to annotations for which chemical standards were not present in an in-house database and by comparison of the experimental MS/MS spectra with those available in the public spectral databases including LIPID MAPS and NIST23.

1.2 Mapping Targeted Feature Extraction Results to the Feature Table

Targeted feature extraction workflows from the originating institutions [1, 4] were used as the ground truth due to their validated high confidence. As annotations were pre-processed independently from the untargeted feature table, the annotations needed to be matched to the feature table by comparing differences in *m/z* and retention time (rt). For the AddNeuroMed-LNEG assay, a 5 *s* rt window and 0.0001 Da tolerance were applied; for AddNeuroMed-LPOS, 15 sescond and 0.005 Da. All annotations that had multiple matches within these windows were excluded from further analysis to retain a high confidence. In the Metformin-HIIE dataset, rt shift was observed during isocratic segment I2 (3.5–10 min) and gradient segment G2 (10–11 min). RT shift was estimated by bootstrapping median rt differences of multiple matches in 60 *s* intervals (10 s rolling window) and modeled with LOWESS regression. A 30 *s* adjusted rt window and $\pm$ 10 ppm mass tolerance were then applied. As multiple matches occurred more frequently in this dataset, they were resolved using the Hungarian algorithm [5] to minimize the linear sum of *m/z* and adjusted rt differences.

1.3 Metformin high-intensity interval exercise (Metformin-HIIE) study dataset

Feature Table:
We used the feature table generated and pre-processed from earlier studies [6, 7]. The untargeted MS1 raw data obtained from UHPLC-ESI(+)-QTOF-MS analysis underwent a data-cleaning process to remove background interference and irrelevant ions. This cleaning was performed via a recursive analysis using MassHunter Profinder software (version B.10.0.2, Agilent Technologies, Santa Clara, CA, USA). Initially, the Molecular Feature Extraction (MFE) algorithm performed chromatographic deconvolution to build all the mass spectral data features, which are the sum of coeluting ions that are related by charge-state envelope, isotopologue pattern, and/or the presence of different adducts and dimers in the analyzed samples. Simultaneously, the MFE aligned these molecular features across all study samples using their mass and rt to create a unified spectrum for each compound group. Subsequently, the MFE

results were employed for Recursive Feature Extraction. The Batch Find by Ion extraction (FbI) algorithm utilized the median mass, median rt, and a composite spectrum derived from the aligned features to enhance reliability. For the present analysis, only the ESI(+) dataset was used, comprising 1,171 features. To identify coeluting adducts of the same feature, the following adducts were selected: [M+H]+, [M+Na]+, [M+K]+, and [M+NH4]+. The neutral loss of water [M+H-H2O]+ was also considered. The results were then reviewed and a manual inspection was performed of all the extracted features and reintegrated them by hand to ensure accuracy. After integration, a .csv file was obtained and exported into Excel with a final list of 1,171 features for futher processing containing monoisotopic mass, rt and abundances.

Targeted Feature Extraction:

Additionally, lipid annotations were obtained using a Human Plasma NIST® SRM® 1950 lipidomic database as a template to perform a protocol described by Martínez et al, [4] with a resulting 131 features mapping to the feature table with lipid subclass specificity (section S.1.2). Briefly, a targeted peak extraction was carried out based on a molecular formula, monoistopic mass, isotope pattern, charge, adduct profile and rt-match approach using the Batch Targeted Feature Extraction (TFE) algorithm of MassHunter Profinder software (B. 10.0.2, Agilent Technologies, Santa Clara, USA). The algorithm employs a .csv file input file containing lipid ID, molecular formula, monoistopic mass and rt information to extract lipids from the MS1 acquisition raw data files using an algorithm referred to as 'Find Compounds by Formula'.

1.4 Estimation of neutral mass candidates for un-grouped feature tables

Allowing multiple ion forms during database matching for all features led to reduced GLC performance compared with searches limited to just [M+H]+ in ESI(+) and [M-H]- in ESI(-). This reduction is likely due to an increased number of false matches, as the number of database queries increases from n (one query per feature when only considering [M+H]+/[M-H]-) to n × m when m alternative ion forms are included. To control this effect, we adopted an approach based on identifying recurrent mass differences among co-eluting ions using kernel density estimation [8, 9]. In this framework, [M+H]+ in ESI(+) and [M-H]- in ESI(-) are always considered, whereas alternative ion forms are included in database matching only when there is evidence supporting their potential presence, as defined by the protocol described below. Furthermore, as an additional filter, we required that the corresponding mass difference had been reported in a curated list of co-eluting mass differences identified across 142 LC–MS studies by Nash et al. [9].

To identify the most common ion forms in the dataset, we examined all pairwise feature *m/z* differences where the features exhibited a positive partial correlation and eluted within one *s* of each other. Gaussian kernel density estimation was applied to the resulting *m/z* differences using a bandwidth of 0.005 to smooth the distribution. Local maxima were then identified using the 'scipy.signal.find_peaks' function, and the full width at half maximum (FWHM) of each peak was used to define the range within which a mass difference was considered potentially associated with a specific ion form. To facilitate interpretation, observed *m/z* difference peaks were compared against a reference table of known electrospray ionisation-derived mass differences curated found in Supplementary table 3 of a paper by Nash et al. [9] and only *m/z* differences that were also in this table were considered.

For the AddNeuroMed-LPOS assay, analysis of the pairwise mass-to-charge (*m/z*) differences revealed prominent peaks corresponding to known adduct and fragment ion forms, suggesting their prevalence in the dataset. Specifically, peaks were observed for *m/z* differences consistent with the [M+H]+, [M+Na]+, [M+K]+, and [M+NH_4]+ charged adducts, as well as ion fragments from in-source fragmentation (ISF) such as [M+H -H_2O]+, [M+H - C_2H_2]+, and [M+H–H_2]+ (**Figure S1** and **Table S2**).

For the AddNeuroMed-LNEG assay, analysis suggested the presence of [M-H]-, ISF ions [M-H - $C_3H_6O_2$]-, [M-H - C_2H_2]- and the neutral adducts [M-H + CH_3COOH]- and clusters [M-H +CH_3COOK]- (**Figure S2** and **Table S3**).

To minimize false positives during accurate mass searches against the database, only the [M+H]+ ion form in positive ionisation mode and the $[M–H]^-$ ion form in negative mode were used as default search forms. The alternative ion forms were only considered if the corresponding feature was associated with a *m/z* difference peak, indicating the likely presence of a specific adduct, cluster or ISF. Additionally, 13C isotopes were considered according to the *m/z* difference peak corresponding to the theoretical mass

difference of 1.0034.

Annotation	m/z difference (theoretical)	mz_centre	mzmin	mzmax	n_edges
Carbon (12C and 13C; M+1)	1.0034	1.004	0.9979	1.0103	1782
H_2	2.0157	2.0086	2.0014	2.0172	401
`[M+NH4]+ - [M+Na]+ difference`	4.9554	4.9566	4.9512	4.9622	323
`[M+Na]+ - [M+39K]+ difference`	15.9739	15.9745	15.97	15.9806	221
C_2H_2	26.0157	26.0168	26.0101	26.024	173
`[M+H]+ - [M+Na]+ difference`	21.9819	21.9832	21.9774	21.9911	169
H_2O (water)	18.0106	18.0113	18.005	18.0164	86

Table S2: AddNeuroMed LPOS m/z peak differences for selected ion forms

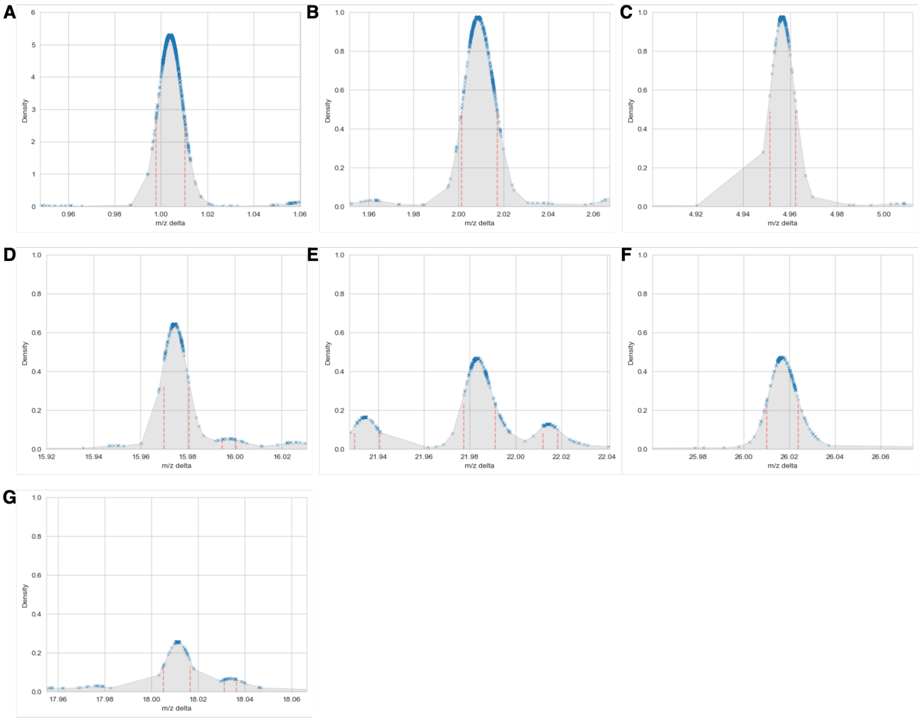

Figure S1: **AddNeuroMed LPOS *m/z* difference peaks** Selected *m/z* difference peaks for the AddNeuroMed LPOS asssay.Red dashed lines correspond to full-width half maximum rounded to nearest observation. Potential *m/z* differences corresponding to the peaks include: **A** Carbon (12C and 13C; M+1) **B** H_2 **C** [M+NH4] - [M+Na] difference **D** $[M+Na]^+$ - $[M+39K]^+$ difference **E** $[M+H]^+$ - $[M+Na]^+$ difference **F** C_2H_2 **G** H_2O (water)

.

Annotation	m/z difference (theoretical)	mz_centre	mzmin	mzmax	n_edges
Carbon (12C and 13C; M+1)	1.0034	1.0031	0.9970	1.0083	758
$C_3H_6O_2$	74.0368	74.0365	74.0309	74.0398	130
CH_3COOK (potassium (39K) acetate)	97.9770	97.9770	97.9718	97.9799	103
C_2H_2	26.0157	26.0157	26.0117	26.0197	59
CH_3COOH (acetic acid)	60.0210	60.0210	60.0169	60.0222	43

Table S3: AddNeuroMed LNEG *m/z* peak differences for selected ion forms

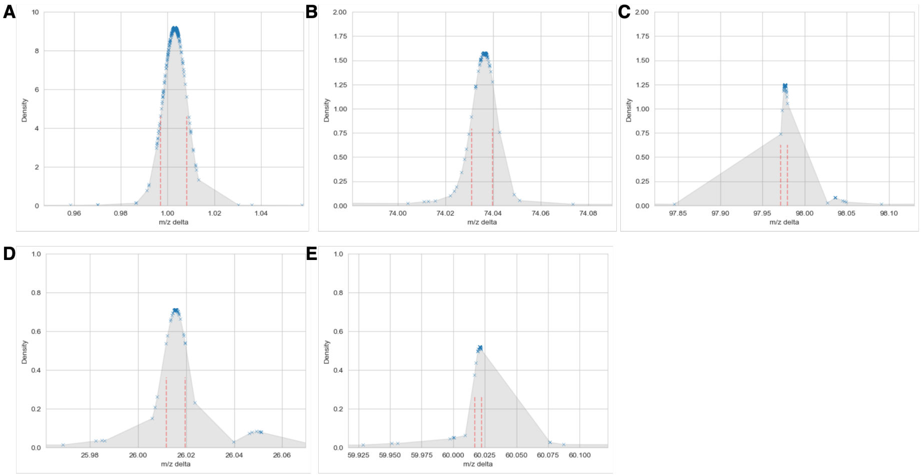

Figure S2: **AddNeuroMed-LNEG *m/z* difference peaks** Selected *m/z* difference peaks for the AddNeuroMed LNEG asssay.Red dashed lines correspond to full-width half maximum rounded to nearest observation. Peaks may not appear smooth as the area under the curve is not from the kernel density estimate but is a linear line to the next observation. Potential *m/z* differences corresponding to the peaks include: **A** Carbon (12C and 13C; M+1) **B** $C_3H_6O_2$ **C** CH_3COOK (potassium (39K) acetate) difference **D** C_2H_2 **E** CH_3COOH (acetic acid)

1.5 LogP Filtering of Database Matches

In the tentative mapping of features to candidate structures in the LMSD, features can map to multiple potential structures. Across the three datasets, the average feature maps to 11.56, 5.66 and 5.51 structures for the AddNeuroMed-LPOS, AddNeuroMed-LNEG and Metformin-HIIE datasets, respectively. Given the chromatic behavior of reversed-phase (RP) columns in untargeted lipidomics, more polar analytes are expected to elute earlier [10]. Therefore, to filter out extreme feature-structure matches that are inconsistent with the behaviour of the column, we implemented a simple model describing rt as a monotonic increasing function of the logP of candidate structures. This reflects the expectation that LogP increases with elution time. Although this approach is an oversimplification which ignores factors such as the pH of the mobile phase, it is adequate for removing highly implausible feature-structure matches. While more advanced RT-prediction models exist, they typically require annotated training data. To keep our lipid-class prediction model, GLC, annotation-free, we opted for this simple approach to remove structures inconsistent with the chromatographic behavior of the column.

Monotonic regression was done using scikit-learn's [11] IsotonicRegression (default parameters), fitting feature rt values against LMSD-reported logP for candidate structures. Candidate structures with residuals exceeding three standard deviations from the fitted curve were removed. rt and logP values from institutional in-house libraries are shown as blue triangles in **Figure S3**. The AddNeuroMed datasets contributed 99 structures with LIPID MAPS IDs from the National Phenome Centre ROI files (used in the PeakPantheR software [1]). The Metformin-HIIE datasets contributed 279 structures, from the in-house database described by Martínez et al[4]. When a unique LIPID MAPS ID could not be assigned, entries with matching lipid names and molecular formulas in the LMSD were retained, and their logP values were averaged.

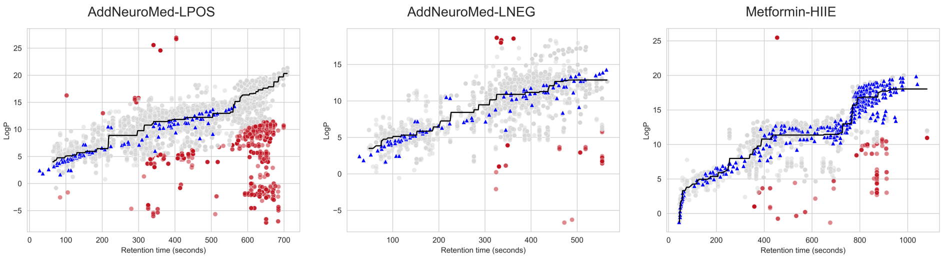

Figure S3: Monotonic regression of LogP from retention time (rt; seconds). Each gray point is the rt of the feature and the LogP of a mapping structure in the LIPID MAPS structural database. The black line is the fitted monotnic regression line. Blue points correspond to the rt and logP of structures from the institutions in-house database for the assay. Red points are feature-structure matches exceeding three standard deviations from the fitted curve.

1.6 The Closest-m/z Model

List of ion forms considered in the feature table to LMSD matching for the AddNeuroMed assays.
LMSD all positive ions list: [M+H]+, [M+H-H2O]+, [M+Na]+, [M+NH4]+, [M+K]+, [M+2H]2+, [M+2Na]2+, [M+2Na-H]+

LMSD all negative ions list: [M-H]-, [M+Cl]-, [M+HCOO]-, [M+CH3COO]-, [M-CH3]-, [M-2H]2-, [M-3H]3-, [M+HCO3]-

Although, [M+Li]+, [M+107Ag]+, [M+109Ag]+ are included in the Lipid Maps bulk structure search under the option 'all ions' for positive mode, these ions were not included in our search as they are not added to the AddNeuroMed mobile phase.

2 Supplementary Results

2.1 Graph Structure Null model

To assess the influence of neighbouring features on GLC lipid class scores, we constructed a GLC null network model by fixing the GGM structure while randomly permuting the assignment of features to nodes. Classification performance metrics (micro F1, macro F1, and overall accuracy) were computed for both lipid subclass and main class levels using the ground truth annotations from the three datasets (AddNeuroMed LPOS, AddNeuroMed LNEG, and Metformin-HIIE). For each metric, the mean and standard error of the mean were estimated across 1000 runs with different random seeds.

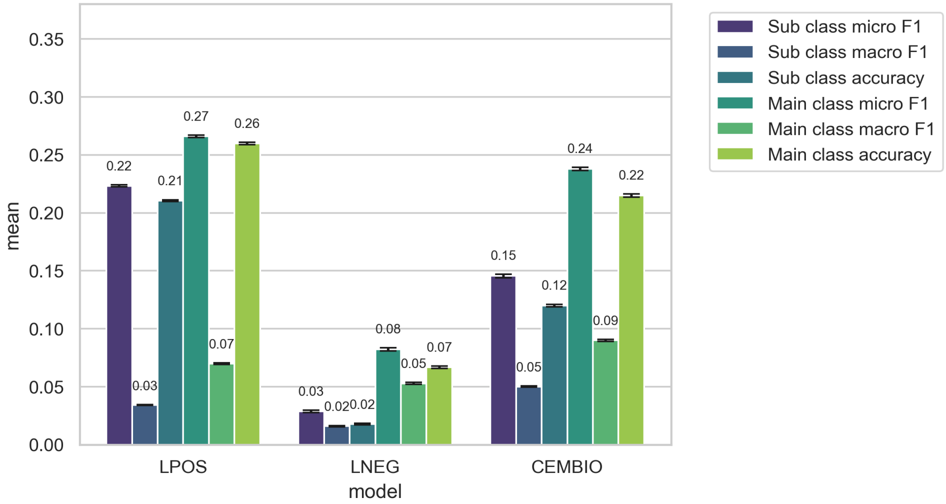

Figure S4: GgmLipidClassifier network null model. Nodes labels in the Gaussian graphical model, corresponding to features, are permuted and GggmLipidClassifer is run. The mean classification performance metrics micro F1, macro F1 and overall accuracy at the LipidMaps subclass and main class are reported for 1000 random seeds. Error bars reflect the standard error of the mean.

2.2 Annotation class clustering in GGM structure

Statistical testing results to evaluate if lipid classes of annotated features are more frequently nearest neighbors in the UMAP embedding of the GGM structure. As outlined in the methods section.

Main class	n	Z-score	P-value	Adjusted P-value
Glycerophosphocholines [GP01]	61	1.711e+01	0.000e+00	0.000e+00
Triradylglycerols [GL03]	59	4.812e+01	0.000e+00	0.000e+00
Phosphosphingolipids [SP03]	13	5.129e+00	1.460e-07	1.460e-06
Fatty esters [FA07]	12	2.138e+01	0.000e+00	0.000e+00
Diradylglycerols [GL02]	9	3.615e+01	0.000e+00	0.000e+00
Glycerophosphoethanolamines [GP02]	7	2.024e+01	0.000e+00	0.000e+00
Neutral glycosphingolipids [SP05]	6	1.819e+01	0.000e+00	0.000e+00
Sterols [ST01]	5	1.029e+01	0.000e+00	0.000e+00
Ceramides [SP02]	4	1.294e+01	0.000e+00	0.000e+00
Isoprenoids [PR01]	4	≤ -10	1.000e+00	1.000e+00

Table S4: AddNeuroMed-LPOS LipidMaps main class annotation clustering statistics.

Subclass	n	Z-score	P-value	Adjusted P-value
Triacylglycerols [GL0301]	59	4.812e+01	0.000e+00	0.000e+00
Diacylglycerophosphocholines [GP0101]	26	8.669e+00	0.000e+00	0.000e+00
1-alkyl,2-acylglycerophosphocholines [GP0102]	13	5.129e+00	1.460e-07	1.898e-06
Ceramide phosphocholines (sphingomyelins) [SP0301]	13	5.129e+00	1.460e-07	1.898e-06
Monoacylglycerophosphocholines [GP0105]	13	6.936e+00	2.012e-12	2.615e-11
Fatty acyl carnitines [FA0707]	12	2.138e+01	0.000e+00	0.000e+00
Diacylglycerols [GL0201]	9	3.615e+01	0.000e+00	0.000e+00
Monoalkylglycerophosphocholines [GP0106]	7	1.067e+01	0.000e+00	0.000e+00
Simple Glc series [SP0501]	6	1.819e+01	0.000e+00	0.000e+00
Diacylglycerophosphoethanolamines [GP0201]	4	2.275e+01	0.000e+00	0.000e+00
Steryl esters [ST0102]	4	1.294e+01	0.000e+00	0.000e+00
C40 isoprenoids (tetraterpenes) [PR0107]	3	≤ -10	1.000e+00	1.000e+00
N-acylsphingosines (ceramides) [SP0201]	3	1.866e+01	0.000e+00	0.000e+00

Table S5: AddNeuroMed-LPOS LipidMaps subclass annotation clustering statistics.

Main class	n	Z-score	P-value	Adjusted P-value
Fatty Acids and Conjugates [FA01]	21	1.598e+01	0.000e+00	0.000e+00
Acidic glycosphingolipids [SP06]	12	1.053e+01	0.000e+00	0.000e+00
Glycerophosphoethanolamines [GP02]	11	7.829e+00	2.442e-15	1.221e-14
Glycerophosphates [GP10]	9	9.034e+00	0.000e+00	0.000e+00
Glycerophosphoinositols [GP06]	7	1.083e+01	0.000e+00	0.000e+00

Table S6: AddNeuroMed-LNEG LipidMaps main class annotation clustering statistics.

Subclass	n	Z-score	P-value	Adjusted P-value
Unsaturated fatty acids [FA0103]	16	2.303e+01	0.000e+00	0.000e+00
Sulfoglycosphingolipids (sulfatides) [SP0602]	12	1.053e+01	0.000e+00	0.000e+00
Diacylglycerophosphoethanolamines [GP0201]	7	5.228e+00	8.584e-08	6.009e-07
Monoacylglycerophosphoinositols [GP0605]	7	1.083e+01	0.000e+00	0.000e+00
Monoacylglycerophosphates [GP1005]	6	1.603e+01	0.000e+00	0.000e+00
Straight chain fatty acids [FA0101]	4	1.282e+01	0.000e+00	0.000e+00
Monoacylglycerophosphoethanolamines [GP0205]	3	9.538e+15	0.000e+00	0.000e+00

Table S7: AddNeuroMed-LNEG LipidMaps subclass annotation clustering statistics.

Main class	n	Z-score	P-value	Adjusted P-value
Glycerophosphocholines [GP01]	99	6.698e+00	1.056e-11	9.504e-11
Ceramides [SP02]	41	7.974e+00	7.772e-16	6.994e-15
Triradylglycerols [GL03]	22	9.113e+00	0.000e+00	0.000e+00
Glycerophosphoethanolamines [GP02]	17	1.735e+00	4.139e-02	3.725e-01
Fatty esters [FA07]	12	4.555e+01	0.000e+00	0.000e+00
Sterols [ST01]	8	3.876e+00	5.319e-05	4.787e-04
Diradylglycerols [GL02]	6	≤ -10	1.000e+00	1.000e+00
Glycerophosphoinositols [GP06]	6	1.381e+01	0.000e+00	0.000e+00
Neutral glycosphingolipids [SP05]	3	≤ -10	1.000e+00	1.000e+00

Table S8: Metformin-HIIE LipidMaps main class annotation clustering statistics.

Subclass	n	Z-score	P-value	Adjusted P-value
Diacylglycerophosphocholines [GP0101]	48	3.527e+00	2.101e-04	2.521e-03
Triacylglycerols [GL0301]	22	6.629e+00	1.692e-11	2.030e-10
Monoacylglycerophosphocholines [GP0105]	16	6.869e+00	3.228e-12	3.874e-11
N-acylsphingosines (ceramides) [SP0201]	14	3.671e+00	1.208e-04	1.449e-03
Fatty acyl carnitines [FA0707]	12	3.737e+01	0.000e+00	0.000e+00
Steryl esters [ST0102]	8	2.698e+00	3.490e-03	4.188e-02
Diacylglycerols [GL0201]	6	≤ -10	1.000e+00	1.000e+00
Diacylglycerophosphoethanolamines [GP0201]	6	4.208e+00	1.290e-05	1.547e-04
Diacylglycerophosphoinositols [GP0601]	6	1.136e+01	0.000e+00	0.000e+00
Monoacylglycerophosphoethanolamines [GP0205]	5	≤ -10	1.000e+00	1.000e+00
N-acylsphinganines (dihydroceramides) [SP0202]	5	≤ -10	1.000e+00	1.000e+00
Simple Glc series [SP0501]	3	≤ -10	1.000e+00	1.000e+00

Table S9: Metformin-HIIE LipidMaps subclass annotation clustering statistics.

2.3 Number of samples and GLC performance

The impact of sample size on GLC performance was evaluated using the AddNeuroMed-LPOS dataset. For each sample size, ten random subsamples were drawn, a GGM was estimated, and lipid-class predictions were generated using GLC. The median performance across subsamples was recorded, along with the observed minimum–maximum range. As GLC only provides lipid-class predictions for features within the main subgraph of the GGM, both the number of features contained in the main subgraph and the number of features with ground-truth annotations used for performance evaluation were also recorded.

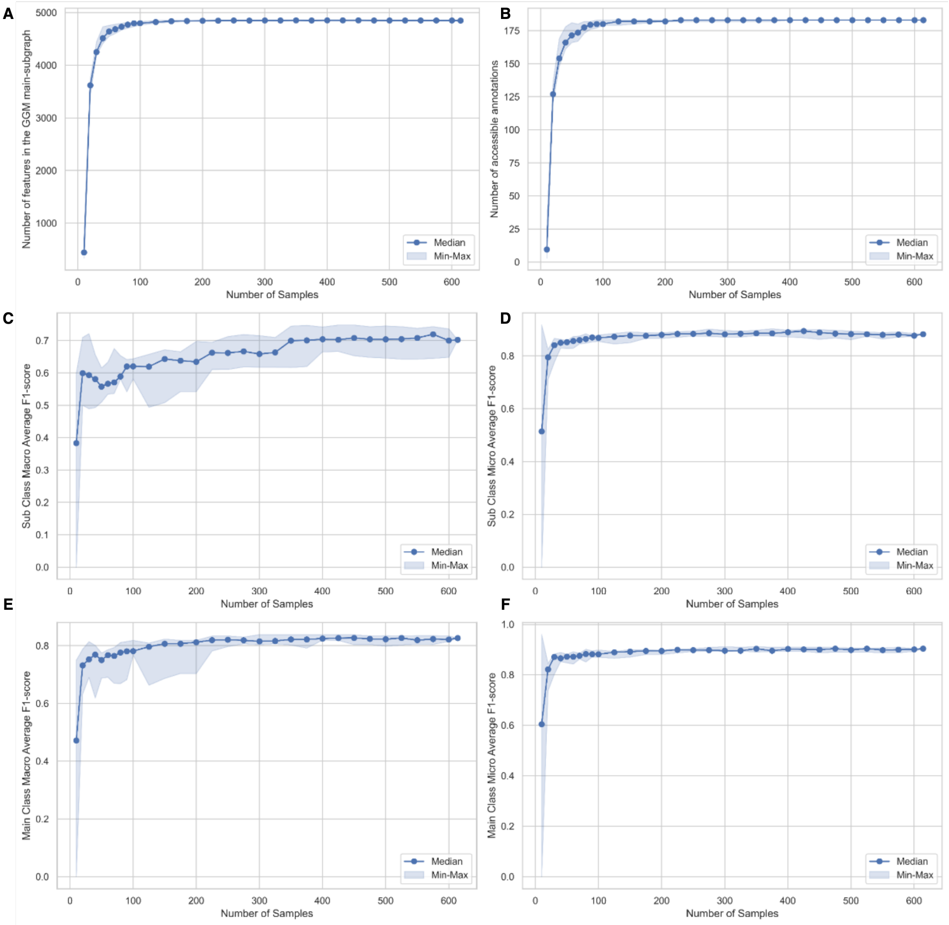

Figure S5: Impact of sample size on Gaussian Graphical Model (GGM) estimation and GgmLipidClassifier (GLC) performance in the AddNeuroMed-LPOS dataset. For each tested sample size, ten random subsamples were generated, and the median values across subsamples was plotted. Shaded regions represent the minimum–maximum (Min–Max) range. Performance metrics were computed using ground-truth lipid annotations available within the GGM main subgraph.**(A)** Number of features included in the GGM main subgraph.**(B)** Number of annotated lipids within the GGM main subgraph used for performance evaluation. **(C)** Lipid subclass macro-averaged F1-score. **(D)** Lipid subclass micro-averaged F1-score. **(E)** Lipid main class macro-averaged F1-score.**(F)** Lipid main class micro-averaged F1-score.

2.4 Subclass Prediction clustering in GGM structure

Main class	n	Z-score	P-value	Adjusted P-value
Triacylglycerols [GL0301]	1620	1.098e+02	0.000e+00	0.000e+00
Diacylglycerophosphocholines [GP0101]	1037	3.871e+01	0.000e+00	0.000e+00
Ceramide phosphocholines (sphingomyelins) [SP0301]	327	3.747e+01	0.000e+00	0.000e+00
Monoacylglycerophosphocholines [GP0105]	317	5.944e+01	0.000e+00	0.000e+00
1-alkyl,2-acylglycerophosphocholines [GP0102]	302	3.628e+01	0.000e+00	0.000e+00
N-acylsphingosines (ceramides) [SP0201]	123	6.012e+01	0.000e+00	0.000e+00
Ceramide phosphoethanolamines [SP0302]	95	8.067e+01	0.000e+00	0.000e+00
Simple Glc series [SP0501]	88	9.178e+01	0.000e+00	0.000e+00
Diacylglycerols [GL0201]	75	1.566e+02	0.000e+00	0.000e+00
Diacylglycerophosphoinositols [GP0601]	66	4.051e+01	0.000e+00	0.000e+00
Vitamin D3 and derivatives [ST0302]	66	3.885e+01	0.000e+00	0.000e+00
1-acyl,2-alkylglycerols [GL0207]	56	2.828e+01	0.000e+00	0.000e+00
1-(1Z-alkenyl),2-acylglycerophosphocholines [GP0103]	55	1.325e+01	0.000e+00	0.000e+00
Fatty acyl carnitines [FA0707]	54	1.284e+02	0.000e+00	0.000e+00
Cholesterol and derivatives [ST0101]	51	1.430e+01	0.000e+00	0.000e+00
Ergosterols and C24-methyl derivatives [ST0103]	48	7.432e+01	0.000e+00	0.000e+00
1-(1Z-alkenyl),2-acylglycerophosphoethanolamines [GP0203]	43	4.967e+01	0.000e+00	0.000e+00
1-alkyl,2-acylglycerophosphoserines [GP0302]	32	1.608e+01	0.000e+00	0.000e+00
Monoacylglycerophosphoethanolamines [GP0205]	29	7.125e+01	0.000e+00	0.000e+00
Monoalkylglycerophosphocholines [GP0106]	27	3.629e+01	0.000e+00	0.000e+00
1-alkyl,2-acylglycerophosphoethanolamines [GP0202]	26	3.725e+01	0.000e+00	0.000e+00
C40 isoprenoids (tetraterpenes) [PR0107]	25	1.384e+02	0.000e+00	0.000e+00
Oxidized glycerophosphoinositols [GP2005]	24	5.323e+01	0.000e+00	0.000e+00
Ceramide 1-phosphates [SP0205]	23	4.062e+01	0.000e+00	0.000e+00
1-deoxyceramides [SP0206]	23	3.108e+01	0.000e+00	0.000e+00
N-acylsphinganines (dihydroceramides) [SP0202]	19	2.021e+02	0.000e+00	0.000e+00
Fatty alcohols [FA05]	17	3.159e+01	0.000e+00	0.000e+00
Fatty acid estolides [FA0709]	16	≤ -10	1.000e+00	1.000e+00

Table S10: UMAP nearest-neighbor clustering results on GgmLipidClassifer subclass predictions (subclass represented by ≥ 15 predictions) for the AddNeuroMed-LPOS dataset.

2.5 RT-MZ plots AddNeuroMed-LPOS dataset

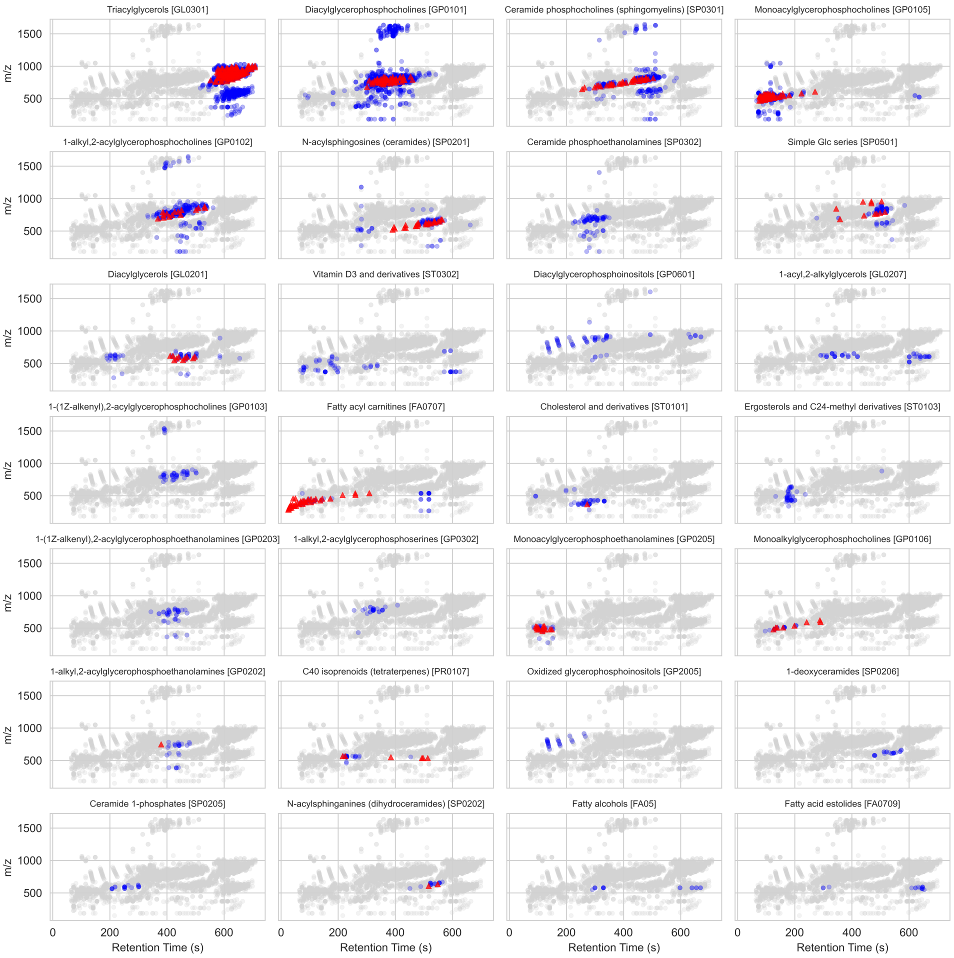

Figure S6: m/z–retention time plots show all detected features in grey, GgmLipidClassifer-predicted lipids in blue, and lipids present in the National Phenome Center in-house database in red. Results shown for all subclasses represented by ≥ 15 predictions for the AddNeuroMed-LPOS dataset.

2.6 Quality score statistical testing

dataset	variable	statistic	P-value	P-adj
AddNeuroMed-LNEG	lsi-score	6.045e+02	6.751e-03	6.076e-02
AddNeuroMed-LNEG	pcor-score	6.650e+02	3.995e-04	3.596e-03
AddNeuroMed-LNEG	product-score	7.110e+02	2.501e-05	2.251e-04
AddNeuroMed-LPOS	lsi-score	3.062e+03	2.092e-05	1.882e-04
AddNeuroMed-LPOS	pcor-score	3.318e+03	3.355e-07	3.020e-06
AddNeuroMed-LPOS	product-score	3.365e+03	1.222e-07	1.100e-06
metformin-HIIE	lsi-score	6.010e+03	7.685e-06	6.917e-05
metformin-HIIE	pcor-score	6.046e+03	5.730e-06	5.157e-05
metformin-HIIE	product-score	6.417e+03	3.120e-08	2.808e-07

Table S11: Quality scores for GLC predictions with ground-truth annotations are stratified by whether the LMSD subclass was correctly predicted. Scores include the Local Simpson's Index (LSI), the partial correlation score (PCOR), and the product of LSI and PCOR. Correctly and incorrectly predicted annotations are tested with the Mann-Whitney U test, and p-values are adjusted by the Bonferroni test.

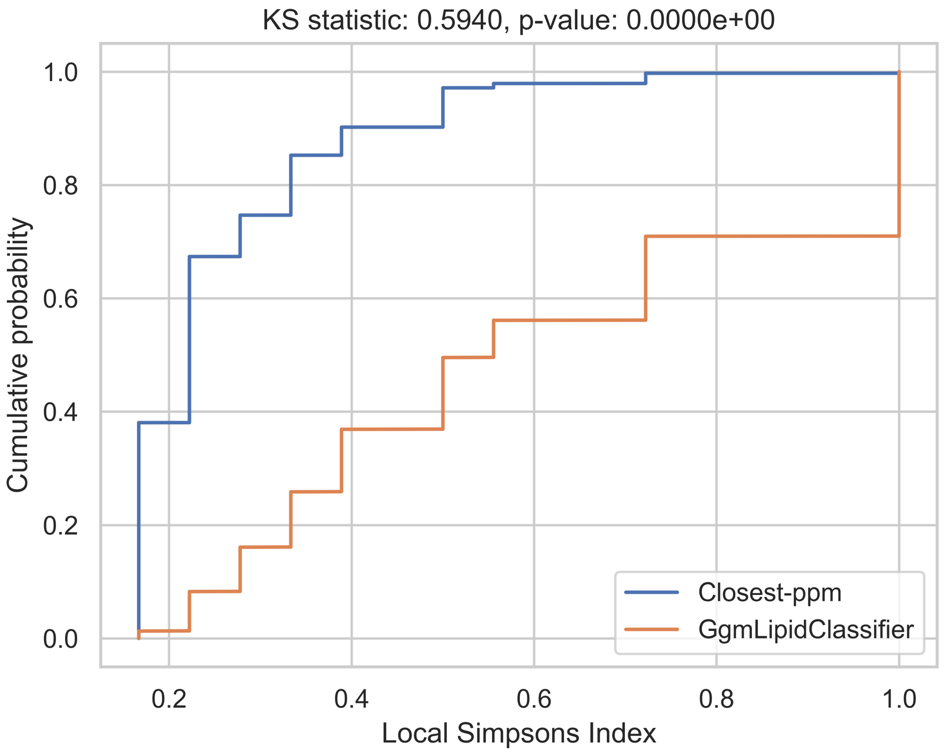

Figure S7: Comparison of Local Simpson's Index (LSI) between the closest-m/z baseline model and GgmLipidClassifier predictions on the AddNeuroMed-LPOS dataset. LSI scores for the closest-m/z model were aggregated over 100 runs with different random seeds. Only features for which the closest-textitm/z model could make predictions for where considered. The plot shows the cumulative density function (CDF) of LSI scores. Differences between distributions were assessed using the Kolmogorov–Smirnov test.

2.7 Lipid Subclass Enrichment Analysis

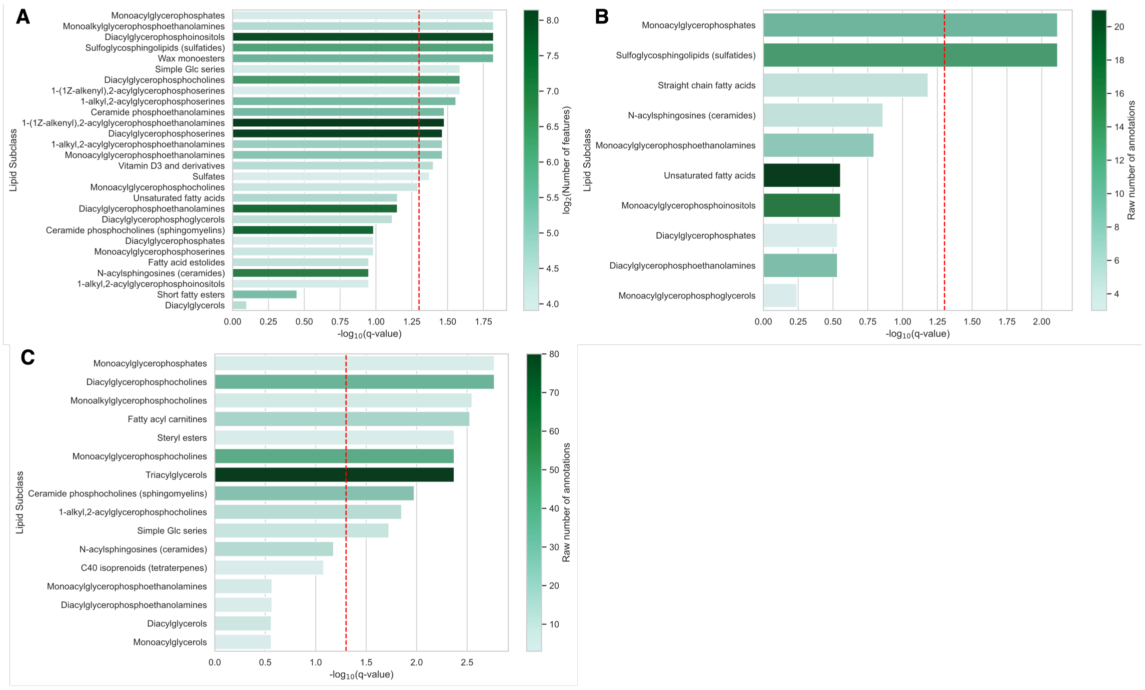

Figure S8: LMSD subclass enrichment between Alzheimer's disease (AD) and controls (CTL) was assessed using an empirical Fisher's method, with Benjamini–Hochberg adjusted p-values shown for each class; the red line indicates an adjusted p-value of 0.05. **(A)** shows results for GgmLipidClassifier predictions in the AddNeuroMed-LNEG dataset, with the color bar representing the log-two number of predicted features per subclass. **(B–C)** show results from targeted feature extraction annotations in negative and positive ionization modes, respectively, for the AddNeuroMed datasets. The color bar indicates the number of annotated features in each subclass.

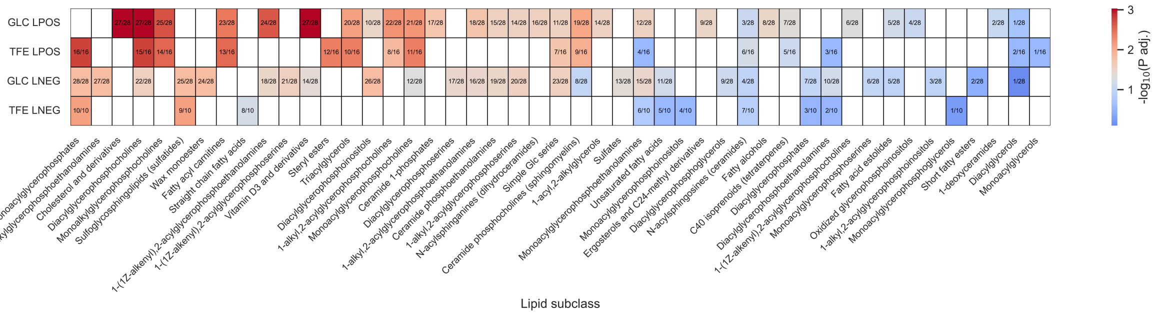

Figure S9: LMSD subclass enrichment between Alzheimer's disease and controls in the AddNeuroMed datasets. Each row corresponds to an analysis using GgmLipidClassifier (GLC) predictions for AddNeuroMed-LPOS (GLC LPOS) and AddNeuroMed-LNEG (GLC LNEG), or targeted feature extraction algorithm (TFE) annotations in ESI(+/-). Lipid subclasses are ordered by the mean adjusted p-value across analyses in which the subclass was sufficiently detected ($\geq$ 15 features for GLC, $\geq$ 3 for TFE). Cells are labeled by their ranking within each analysis and colored according to their $\log_{10}$(adjusted P-value).

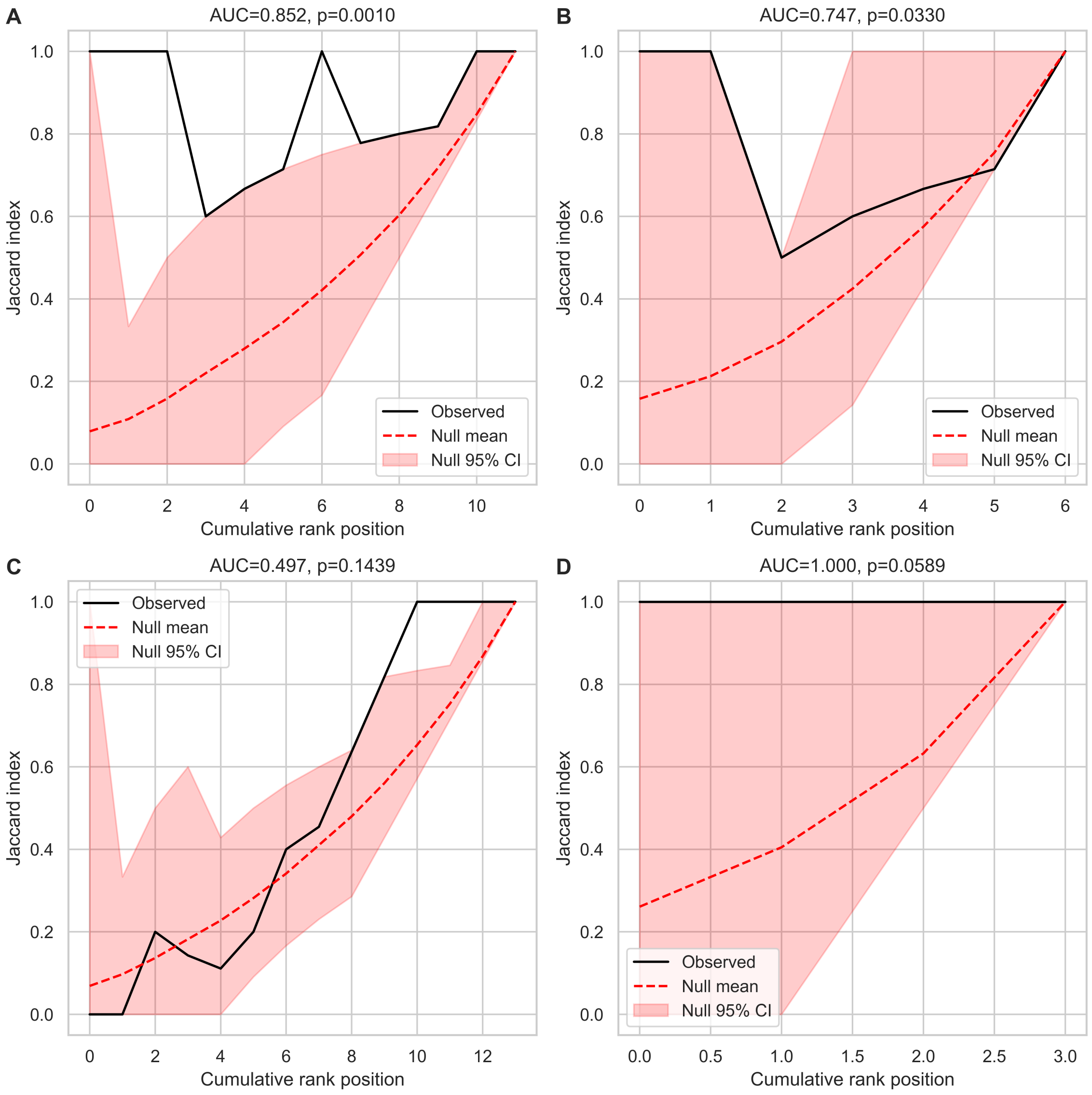

Figure S10: Cumulative Jaccard similarity between significance ranking for (A) GgmLipidClassifier (GLC) predictions and annotations on the AddNeuroMed-LPOS dataset, (B) GLC predictions and annotations in the AddNeuroMed-LNEG dataset, (C) GLC predictions across ESI(+/-) modes, and (D) annotations across ionization modes. The black line shows the observed cumulative Jaccard index. The red line indicates the mean null curve from 1,000 random rankings. The shaded area represents the 95% confidence interval of the null distribution. The normalized area under the curve (AUC) for the observed similarity is shown in the title and empirical p-value from comparing the observed similarity to the null. Higher curves indicate stronger concordance between rankings.

References

[1] Arnaud M Wolfer, Gonçalo D S Correia, Caroline J Sands, Stephane Camuzeaux, Ada H Y Yuen, Elena Chekmeneva, Zoltán Takáts, Jake T M Pearce, and Matthew R Lewis. peakPantheR, an R package for large-scale targeted extraction and integration of annotated metabolic features in LC–MS profiling datasets. *Bioinformatics*, 37(24):4886–4888, June 2021.

[2] Matthew Lewis, Elena Chekmeneva, Stephane Camuzeaux, Caroline Sands, Ada Yuen, Mark David, Ash Salam, Katie Chappell, Benjamin Cooper, Gordon Haggart, Lynn Maslen, María Gómez-Romero, Verena Horneffer-van der Sluis, Gonçalo Correia, and Zoltan Takats. An Open Platform for Large Scale LC-MS-Based Metabolomics. *ChemRxiv*, 2022.

[3] Lloyd W. Sumner, Alexander Amberg, Dave Barrett, Michael H. Beale, Richard Beger, Clare A. Daykin, Teresa W.-M. Fan, Oliver Fiehn, Royston Goodacre, Julian L. Griffin, Thomas Hankemeier, Nigel Hardy, James Harnly, Richard Higashi, Joachim Kopka, Andrew N. Lane, John C. Lindon, Philip Marriott, Andrew W. Nicholls, Michael D. Reily, John J. Thaden, and Mark R. Viant. Proposed minimum reporting standards for chemical analysis Chemical Analysis Working Group (CAWG) Metabolomics Standards Initiative (MSI). *Metabolomics: Official Journal of the Metabolomic Society*, 3(3):211–221, September 2007.

[4] Sara Martínez, Miguel Fernández-García, Sara Londoño-Osorio, Coral Barbas, and Ana Gradillas. Highly reliable LC-MS lipidomics database for efficient human plasma profiling based on NIST SRM 1950. *Journal of Lipid Research*, 65(11):100671, October 2024.

[5] H. W. Kuhn. The Hungarian method for the assignment problem. *Naval Research Logistics Quarterly*, 2(1-2):83–97, 1955.

[6] Stefanos Nikolaidis, Ioannis Kosmidis, Stylianos Papadopoulos, Artemis Lioupi, Marita Gandanidou, Helen Gika, Aristides Dokoumetzidis, Georgios Theodoridis, and Vassilis Mougios. Effect of high-intensity interval exercise on metformin pharmacokinetics in healthy men, assessed through a population pharmacokinetic model. *British Journal of Pharmacology*, September 2025.

[7] René Neuhaus, Thomas Meikopoulos, Ana Gradillas, Carolina Gonzalez-Riaño, Georgios Theodoridis, Stefanos Nikolaidis, Vassilis Mougios, Coral Barbas, Helen Gika, and Alma Villaseñor. A Lipidomic Exploration of the Effects of High-Intensity Interval Exercise in Healthy Men after Metformin Intake. *Journal of Proteome Research*, 24(11):5696–5709, November 2025.

[8] Nathaniel G. Mahieu and Gary J. Patti. Systems-Level Annotation of a Metabolomics Data Set Reduces 25000 Features to Fewer than 1000 Unique Metabolites. *Analytical Chemistry*, 89(19):10397–10406, October 2017.

[9] William J. Nash, Judith B. Ngere, Lukas Najdekr, and Warwick B. Dunn. Characterization of Electrospray Ionization Complexity in Untargeted Metabolomic Studies. *Analytical Chemistry*, 96(27):10935–10942, June 2024.

[10] Magdaléna Ovčačíková, Miroslav Lísa, Eva Cífková, and Michal Holčapek. Retention behavior of lipids in reversed-phase ultrahigh-performance liquid chromatography–electrospray ionization mass spectrometry. *Journal of Chromatography A*, 1450:76–85, June 2016.

[11] Fabian Pedregosa, Gaël Varoquaux, Alexandre Gramfort, Vincent Michel, Bertrand Thirion, Olivier Grisel, Mathieu Blondel, Peter Prettenhofer, Ron Weiss, Vincent Dubourg, Jake Vanderplas, Alexandre Passos, David Cournapeau, Matthieu Brucher, Matthieu Perrot, and Édouard Duchesnay. Scikit-learn: Machine Learning in Python. *Journal of Machine Learning Research*, 12(85):2825–2830, 2011.
